# Upconversion Nanoparticle‐Covalent Organic Framework Core–shell Particles as Therapeutic Microrobots Trackable With Optoacoustic Imaging

**DOI:** 10.1002/adma.202418425

**Published:** 2025-03-07

**Authors:** Dong Wook Kim, Paul Wrede, Andrés Rodríguez‐Camargo, Yi Chen, Nihal Olcay Dogan, Chaim Glück, Bettina V. Lotsch, Daniel Razansky, Metin Sitti

**Affiliations:** ^1^ Physical Intelligence Department Max Planck Institute for Intelligent Systems 70569 Stuttgart Germany; ^2^ Nanochemistry Department Max Planck Institute for Solid State Research 70569 Stuttgart Germany; ^3^ Institute for Biomedical Engineering University of Zurich and ETH Zurich Zürich 8093 Switzerland; ^4^ Department of Chemistry University of Stuttgart 70569 Stuttgart Germany; ^5^ Department of Chemistry Ludwig‐Maximilians‐Universität (LMU) 81377 Munich Germany; ^6^ e‐conversion Lichtenbergstrasse 4a 85748 Garching Germany; ^7^ Institute of Pharmacology and Toxicology University of Zürich Zürich 8057 Switzerland; ^8^ School of Medicine and College of Engineering Koç University Istanbul 34450 Turkey

**Keywords:** covalent organic frameworks, imaging contrast agents, microrobots, optoacoustic imaging, upconversion nanoparticles

## Abstract

Despite the development of various medical imaging contrast agents, integrating contrast signal generation with therapeutic and microrobotic functions remains challenging without complicated fabrication processes. In this study, upconversion nanoparticle‐covalent organic framework (UCNP‐COF) core–shell sub‐micron particles are developed that function as therapeutic microrobots trackable with multi‐spectral optoacoustic tomography (MSOT) imaging and can be loaded with desired therapeutic molecular agents in a customizable manner. The mechanism of optoacoustic signal generation in UCNP‐COF particles is attributed to the quenching of upconversion luminescence emitted by the UCNPs, which is absorbed by the encapsulating COF and subsequently converted into acoustic waves. Unlike other microparticulate agents previously imaged with MSOT, UCNP‐COF particles do not pose concerns about their stability and biocompatibility. Simultaneously, the mesoporous texture of the COF provides a large surface area, allowing for the efficient loading of various drug molecules, which can be released at target sites. Furthermore, the magnetic UCNP‐COF Janus particles can be magnetically navigated through in vivo vasculature while being visualized in real‐time with volumetric MSOT. This study proposes an approach to design photonic materials with multifunctionality, enabling high‐performance medical imaging, drug delivery, and microrobotic manipulation toward their future potential clinical use.

## Graphical abstract

Upconversion nanoparticle‐covalent organic framework core–shell particles provide enhanced contrast for optoacoustic imaging by leveraging the optical absorption of upconversion luminescence within the covalent organic framework matrix. Beyond their role as contrast agents, these particles enable customizable therapeutic agent loading and release, as well as magnetic microrobotic steering within vascular environments.

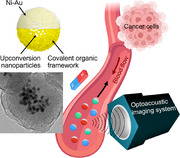

## 1 Introduction

Optoacoustic imaging, also known as photoacoustic imaging, has rapidly gained prominence due to its appealing features as a non‐invasive, multi‐parametric clinical imaging modality^.[^ ^1^, ^2^, ^3^, ^4^, ^5^ ^]^ The method uses nanosecond near‐infrared (NIR) optical pulses to irradiate target biological tissues or contrast agents, leading to thermoelastic expansion in the light‐absorbing targets. This expansion generates broadband acoustic waves, which are subsequently detected by a transducer and can then be used to reconstruct the optical absorption distributions in the tissue.^[^ ^1^, ^2^, ^3^ ^]^ Owing to the weak scattering and superb penetration of acoustic waves into biological tissues, optoacoustic imaging offers high spatial resolution, deep‐tissue imaging capabilities, and an optimized signal‐to‐noise ratio (SNR).^[^ ^4^, ^5^ ^]^ Additionally, entire 3D volumes can be captured in real‐time using a single laser pulse—an advantage not easily achievable with conventional fluorescence or ultrasound imaging.^[^ ^4^, ^5^ ^]^ Furthermore, multi‐spectral optoacoustic tomography (MSOT) provides molecular imaging capabilities by leveraging the distinct optical absorption spectra of various molecular biomarkers, such as lipids, water, collagen, melanin, hemoglobin, and deoxyhemoglobin, to distinguish specific vascular features, tissues, and organs. These distinctive characteristics present significant practical benefits for biological and clinical applications.^[^ ^1^, ^2^, ^3^, ^4^, ^5^ ^]^

Recently, various effective MSOT imaging contrast agents, such as molecular dyes,^[^ ^6^ ^]^ gold nanorods,^[^ ^7^ ^]^ carbon nanomaterials,^[^ ^8^, ^9^ ^]^ quantum dots,^[^ ^8^, ^9^ ^]^ and upconversion nanoparticles,^[^ ^10^ ^]^ have been developed to enhance signal contrast. Among these, Indocyanine Green (ICG), an FDA‐approved diagnostic and therapeutic dye, has been widely used as MSOT contrast agent due to its strong NIR absorption and water solubility.^[^ ^11^ ^]^ However, ICG faces instability issues related to photobleaching, thermal degradation, and aqueous instability.^[^ ^12^, ^13^ ^]^ When exposed to prolonged light during imaging or higher temperatures in aqueous or in vivo environments, ICG molecules can aggregate or undergo irreversible transformations, resulting in a shorter imaging time, making it unsuitable for extended angiographic observation.^[^ ^13^ ^]^

Other metal‐, carbon‐, or lanthanide‐based nanomaterials have gained attention as effective contrast‐generating alternatives to ICG.^[^ ^8^, ^9^ ^]^ These materials have also been combined with ICG or other drug molecules to further enhance imaging contrast or provide therapeutic effects.^[^ ^14^, ^15^, ^16^ ^]^ However, biologically incompatible metal or lanthanide elements raise concerns regarding long‐term metabolism,^[^ ^17^, ^18^ ^]^ whilst the use of ICG still presents stability issues. Additionally, maximizing the loading of therapeutic or functional agents into these nanomaterials to achieve multi‐functionality in a single particle or agent requires complex surface modifications or multiple steps, such as sequential attachment of target agents followed by additional passivation,^[^ ^14^, ^15^, ^16^ ^]^ which complicates the fabrication process. This complexity poses obstacles to incorporating additional capabilities beyond imaging and therapeutic functions, such as microrobotic features that could enable wireless navigation of therapeutic agents to target locations while being tracked via MSOT imaging.^[^ ^19^ ^]^

**Figure 1:**
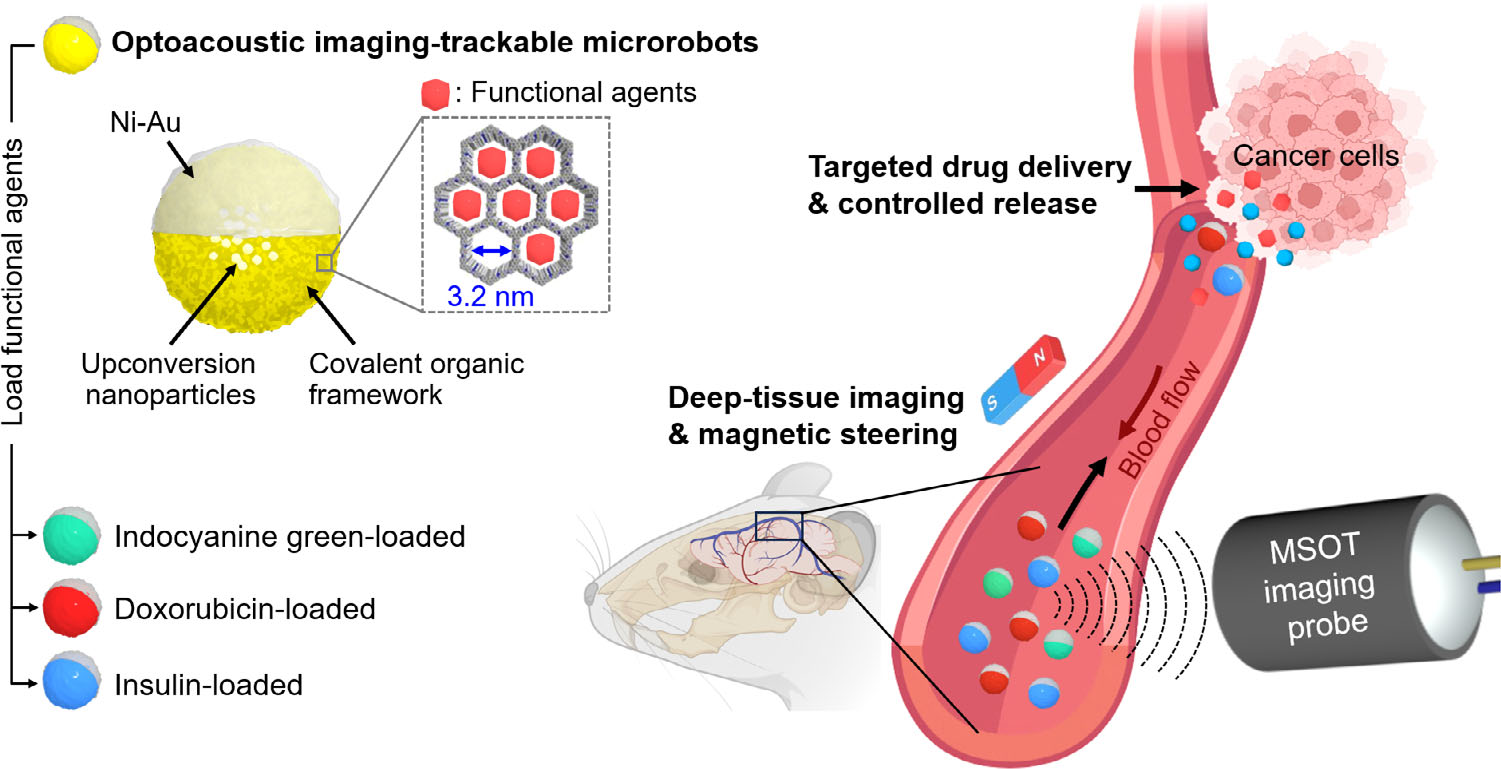
Schematic illustration of multispectral optoacoustic tomography (MSOT) imaging‐trackable therapeutic microrobots. These sub‐micron particles, hybridized with upconversion nanoparticles and covalent organic frameworks, serve as multifunctional contrast agents for deep‐tissue MSOT imaging. They integrate drug‐loading and targeted release capabilities with microrobotic functionality, featuring customizable functional agent loading and magnetic steering.

In this study, we introduce sub‐micron particles hybridized with lanthanide‐doped upconversion nanoparticles (UCNPs) core and covalent organic frameworks (COFs) shell as multifunctional contrast agents for MSOT imaging, with integrated drug‐loading/release capabilities and microrobotic functionality. These UCNP‐COF core–shell particles produce strong MSOT responses with high environmental stability and biocompatibility, while their highly ordered mesopores are ideal for customizable therapeutic loading. In the UCNP‐COF particles, MSOT signal generation occurs when the COF absorbs upconversion luminescence (UCL) emitted by the UCNPs, converting it into a thermoelastic expansion that yields detectable MSOT signals. The mesopores formed by molecular ordering within the COF provide a large surface area with optimal pore size, facilitating the efficient loading of therapeutic or imaging agents. This feature enables controlled drug release at target sites and enhances MSOT contrast beyond what UCNP‐COF particles alone can achieve. Additionally, UCNP‐COF particles can be converted into magnetic Janus microrobots by coating them with a magnetic nanofilm. This transformation enables their magnetic steering within in vivo and in vitro vasculature while carrying therapeutic agents and being tracked in real‐time via 3D MSOT (**Figure** **1**).

## 2 Results and Discussion

### 2.1 Synthesis and Characterization of UCNP‐COF Particles

**Figure 2:**
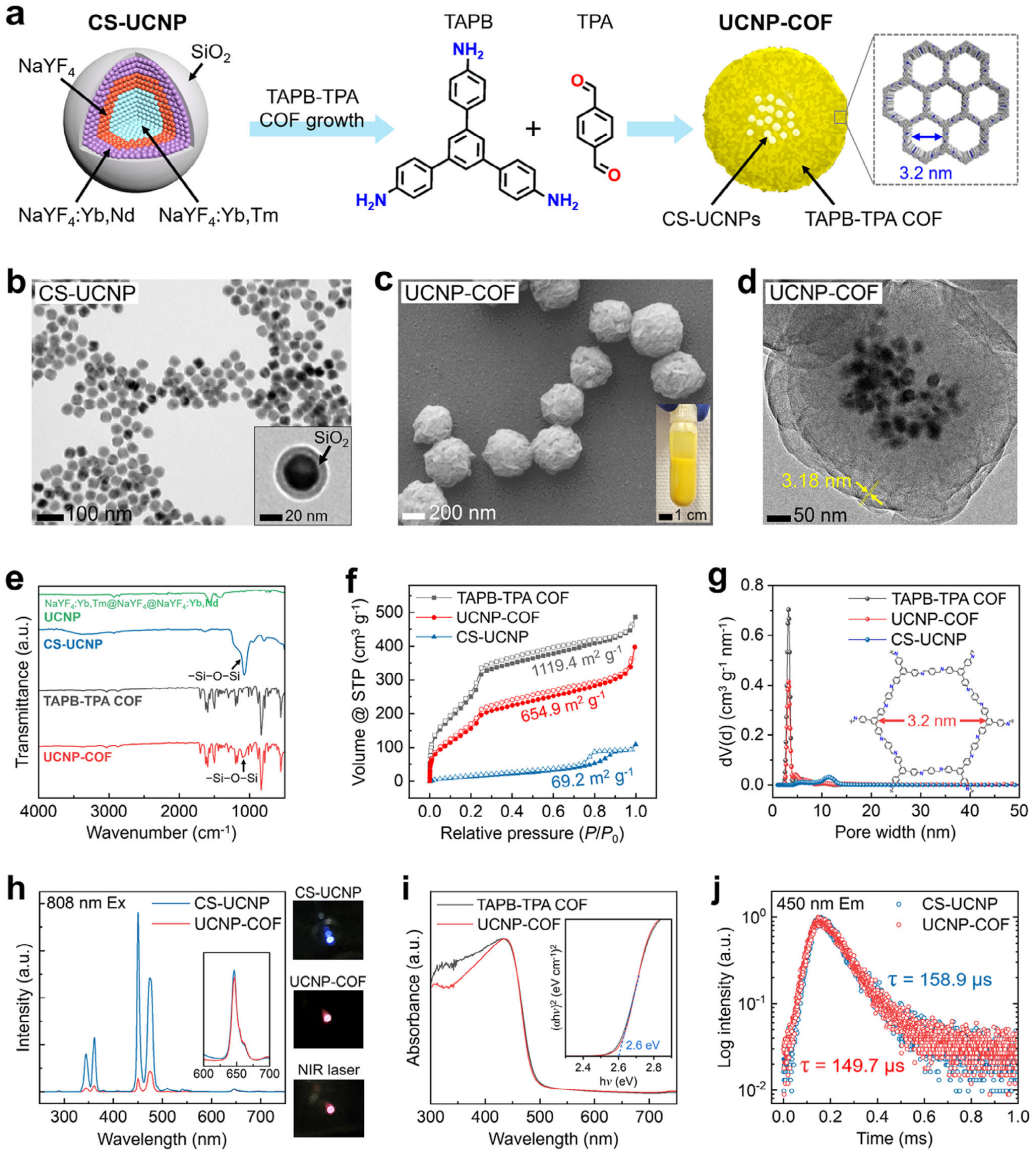
Synthesis and characterization of upconversion nanoparticle‐covalent organic framework (UCNP‐COF) core–shell particles. a) Schematic illustration of the UCNP‐COF particle synthesis process. b) Transmission electron microscopy (TEM) images of CS‐UCNPs. c,d) Scanning electron microscopy (SEM) image (c) and high‐resolution TEM image (d) of UCNP‐COF particles. The inset in (c) shows a photograph of UCNP‐COF particles dispersed in deionized water. e) Fourier‐transform infrared spectroscopy (FT‐IR) spectra of four different samples. f,g) N_2_ adsorption‐desorption isotherms of the different samples measured at standard temperature and pressure (STP) and their calculated surface areas (f) and pore size distributions (g). h) Fluorescence spectra of CS‐UCNPs and UCNP‐COF particles under continuous‐wave 808 nm NIR laser excitation (Ex). The photographs on the right show CS‐UCNPs and UCNP‐COF powder samples under 808 nm laser illumination, along with the bare laser beam. i) Absorption spectra of TAPB‐TPA COF and UCNP‐COF particles. The inset graph shows Tauc plots for both samples to determine their band gap energy. j) Fluorescence decay curves and measured lifetimes at 450 nm emission (Em) for CS‐UCNPs and UCNP‐COF particles.

**Figure** **2**a schematically depicts the synthesis process of UCNP‐COF sub‐micron particles. First, lanthanide‐doped, multi‐layered NaYF_4_:Yb,Tm@NaYF_4_@NaYF_4_:Yb,Nd core UCNPs were synthesized via the solvothermal method.^[^ ^20^ ^]^ Through the sequential deposition of three lanthanide layers–NaYF_4_:Yb,Tm, NaYF_4_, and NaYF_4_:Yb,Nd–core UCNPs with an average diameter of 31.4 ± 2.2 nm were obtained (Figure S1, Supporting Information). Subsequently, uniform silica (SiO_2_) shells ≈8 nm‐thick were coated onto the core UCNPs, forming core–shell UCNPs (CS‐UCNPs) (Figure 2b and Figure S1, Supporting Information). The SiO_2_ shells allow the CS‐UCNPs to be uniformly dispersed in polar solvents during COF formation. Finally, on the CS‐UCNPs dispersed in acetonitrile, 2D TAPB‐TPA imine‐linked covalent organic framework sheets began to grow, with 1,3,5‐tris(4‐aminophenyl)benzene (TAPB) and terephthalaldehyde (TPA) serving as building blocks. These 2D TAPB‐TPA network structures gradually formed around the CS‐UCNPs, ultimately forming 3D spherical UCNP‐COF particles.

As observed in the scanning electron microscopy (SEM) images and digital photographs of the UCNP‐COF particles (Figure 2c and Figure S2, Supporting Information), the particles exhibited a bright yellow color under white light and a narrow size distribution, with an average diameter of 421.0 ± 40.3 nm. High‐resolution transmission electron microscopy (HR‐TEM) images presented in Figure 2d revealed that each UCNP‐COF particle contains multiple CS‐UCNPs at its center, typically ranging from ≈10 to a maximum of 30 CS‐UCNPs per particle (Figure S2, Supporting Information). The COF matrix of the UCNP‐COF particles exhibited a lattice spacing of 3.18 nm, corresponding to the (100) planes, which is consistent with that of pristine TAPB‐TPA COF particles that do not contain CS‐UCNPs (Figure S3, Supporting Information). Additionally, elemental mapping of the UCNP‐COF particles showed a clear distinction between the CS‐UCNPs and the surrounding COF matrix (Figure S4, Supporting Information).

Figure 2e presents the Fourier transform‐infrared (FT‐IR) spectra of the core UCNPs, CS‐UCNPs, TAPB‐TPA COF, and UCNP‐COF particles. The peak corresponding to Si─O─Si stretching at 1100 cm^−1^ was observed only in the CS‐UCNPs, distinguishing them from the core UCNPs. In the TAPB‐TPA COF and UCNP‐COF particles, characteristic peaks at 833 cm^−1^ (aromatic C─H), 1500 cm^−1^ (aromatic C═C), and 1621−1631 cm^−1^ (imine C═N) were identified. Notably, the 1100 cm^−1^ (Si─O─Si stretching) peak was only observed exclusively in the UCNP‐COF particles due to the incorporation of CS‐UCNPs. We further characterized the CS‐UCNPs, TAPB‐TPA COF, and UCNP‐COF particles using X‐ray diffraction (XRD) measurements (Figure S5, Supporting Information). The presence of SiO_2_ shells in the CS‐UCNPs was confirmed by the broad SiO_2_ diffraction signal ≈20°. Additionally, distinct diffraction peaks of the CS‐UCNPs were observed only in the UCNP‐COF particles, alongside the characteristic peaks of the pristine TAPB‐TPA COF.

In Figure 2f,g, we present the Brunauer‐Emmett‐Teller (BET) surface areas and pore size distributions of the different samples, as determined by N_2_ physisorption isotherms. These measurements were performed by analyzing the adsorption and desorption of N_2_ by the sample over a range of relative pressures (*P*/*P*_0_) under standard temperature and pressure (STP) conditions. The CS‐UCNPs exhibited a BET surface area of only 69.2 m^2^ g^−1^, with no distinct porous structures. The TAPB‐TPA COF particles exhibited a substantially larger surface area of 1119.4 m^2^ g^−1^ and a pore width of 3.2 nm, attributed to their highly ordered mesoporous framework. The UCNP‐COF particles exhibited a lower surface area of 654.9 m^2^ g^−1^ compared to that of TAPB‐TPA COF due to the CS‐UCNPs inside, but they retained the same pore width of 3.2 nm as the pristine TAPB‐TPA COF sample.

The UCL spectra of CS‐UCNPs and UCNP‐COF particles under NIR light excitation are shown in Figure 2h. For spectrum measurement, 50 mg of dried CS‐UCNPs and UCNP‐COF particles were collected in a carved aperture on a glass slide, and the spectrum was obtained while a laser beam passed through the sample. Under continuous‐wave NIR (808 nm wavelength) laser irradiation, the CS‐UCNPs emitted ultraviolet (UV) light at 289, 344, and 361 nm, along with strong blue visible emissions at 450 and 474 nm, and weak red emission at 649 nm. These emission wavelengths align well with previous studies on Tm^3+^ and Nd^3+^‐doped UCNPs.^[^ ^20^, ^21^ ^]^ The mechanisms behind the UCL emissions in CS‐UCNPs will be discussed in more detail in the next section. In contrast, UCNP‐COF particles produced substantially reduced relative peak intensities across UV (289, 344, 361 nm) and blue (459, 474 nm) regions compared to CS‐UCNPs, whereas the peak intensity at 649 nm remained nearly unchanged. The pristine TAPB‐TPA COF particles exhibited no detectable luminescence under 808 nm laser irradiation (Figure S6, Supporting Information). The photographs on the right in Figure 2h show the CS‐UCNPs, UCNP‐COF particles, and bare glass film in the path of the 808 nm laser. The photographs were taken without a band‐pass filter, making the 808 nm laser beam visible. The CS‐UCNPs emitted blue‐violet light, but the luminescent color observed in the UCNP‐COF particles showed no significant difference from that of the 808 nm laser beam.

To investigate the diminished UCL emission of UCNP‐COF particles compared to CS‐UCNPs, we measured the absorption spectra and Tauc plots of both TAPB‐TPA COF and UCNP‐COF particles using UV–Vis–NIR spectroscopy (Figure 2i). TAPB‐TPA COF absorbed light at wavelengths below 477 nm, corresponding to an optical band gap (*E*_g_) of 2.6 eV. The absorption spectrum of UCNP‐COF was identical to that of TAPB‐TPA COF, indicating that the encapsulation of CS‐UCNPs with COF does not alter the intrinsic optical properties of the TAPB‐TPA COF. Therefore, in UCNP‐COF particles, the UV and blue UCL emission from the CS‐UCNPs below 477 nm wavelength could be absorbed by the surrounding TAPB‐TPA COF, leading to UCL emission quenching.

To better understand the UCL quenching mechanism in UCNP‐COF, we compared the UCL decay profile and lifetime of CS‐UCNPs and UCNP‐COF particles (Figure 2j). UCL changes in UCNPs conjugated with band gap‐semiconductors occur through two main mechanisms: non‐radiative Förster resonance energy transfer (FRET) and radiative photon absorption (PA).^[^ ^22^, ^23^, ^24^ ^]^ FRET transfers energy from an excited donor to a nearby acceptor without photon emission, affecting luminescence lifetime.^[^ ^22^ ^]^ In contrast, PA involves photon emission by the donor and subsequent absorption by the acceptor, without changing the lifetime.^[^ ^23^ ^]^ Both FRET and PA can coexist between UCNPs and semiconductors in varying proportions depending on the donor‐acceptor proximity.^[^ ^22^, ^23^, ^24^ ^]^

The average lifetime (τ) of the 450 nm UCL emission in CS‐UCNPs and UCNP‐COF particles was 158.9 and 149.7 µs, respectively. The FRET efficiency (*E*_FRET_) is calculated using the equation $E_{\mathrm{FRET}}=1-\frac{\tau_{\mathrm{DA}}}{\tau_{D}}\;\times 100$, where τ_DA_ is the lifetime of a donor (UCNP) in the presence of an acceptor (COF) (for UCNP‐COF particles), and τ_D_ is the lifetime of a donor in the absence of an acceptor (as in CS‐UCNPs). The 450 nm lifetime of UCNP‐COF particles decreased by only 9.2 µs compared to CS‐UCNPs, resulting in an *E*_FRET_ of 5.79%. This low *E*_FRET_ is attributed to the 8 nm‐thick SiO_2_ shell of CS‐UCNPs, which deteriorates FRET while contributing to PA. This finding aligns with the previous work,^[^ ^22^ ^]^ which reported reduced *E*_FRET_ with increased SiO_2_ interlayer thickness between UCNPs and quantum dots. Additionally, the maintained UCL intensity at 649 nm in both CS‐UCNPs and UCNP‐COF particles indicates that PA, rather than FRET, is the primary mechanism of energy transfer.^[^ ^23^ ^]^ If FRET was the major contributor, we would expect a decrease in the 649 nm UCL intensity along with other wavelength peaks.

### 2.2 UCNP‐COF Particles as MSOT Contrast Agents

**Figure 3:**
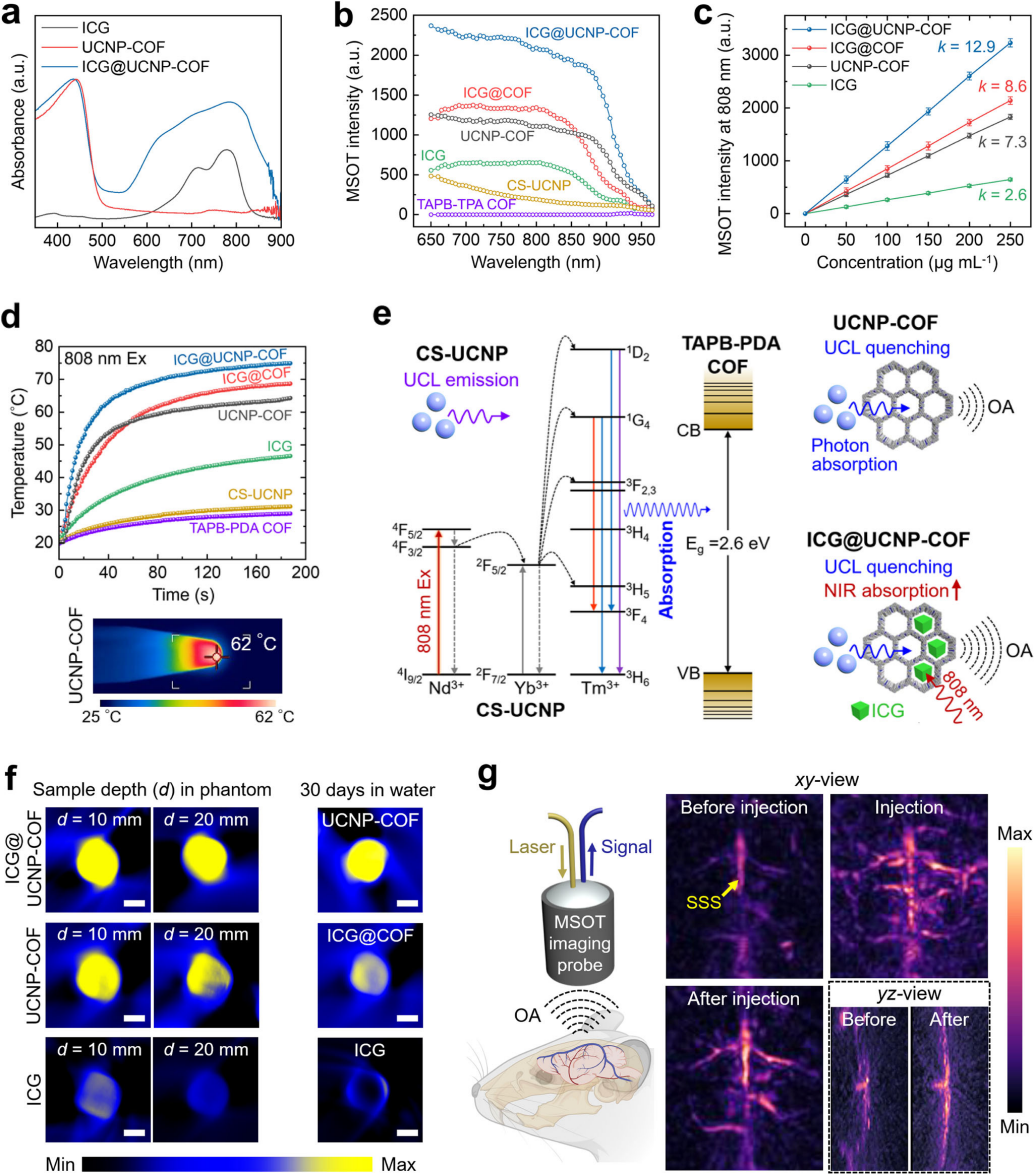
UCNP‐COF particles as MSOT contrast agents. a) Absorption spectra of ICG solution, UCNP‐COF, and ICG@UCNP‐COF particles. b) MSOT spectra of various samples. COF‐based samples were dispersed in agar gel, while CS‐UCNP and ICG samples were prepared in aqueous solution. c) MSOT signal intensity at 808 nm wavelength for different sample concentrations. *k* represents the slope of signal increase. d) Time‐dependent temperature curves of different samples under continuous‐wave 808 nm NIR laser excitation. The infrared image below shows the UCNP‐COF particles under NIR excitation. e) Schematic mechanism of MSOT signal generation from UCNP‐COF and its enhancement in ICG@UCNP‐COF. CB and VB refer to the conduction band and valence band, respectively. f) MSOT imaging of different samples embedded within an agar phantom in different depths (*d*) and after 30 days of aging in water. g) 3D MSOT imaging of brain vasculature before and after injection of UCNP‐COF particles.

Based on previous studies utilizing luminescence quenching by optical absorption as a source of MSOT signal generation,^[^ ^10^, ^25^ ^−^ ^28^ ^]^ UCNP‐COF particles are expected to produce MSOT signals under NIR excitation. To evaluate UCNP‐COF as a contrast agent for MSOT, we compared its performance with a free ICG solution and ICG‐loaded COF particle^[^ ^29^ ^]^ samples. **Figure** **3**a shows the absorption spectra of the ICG solution, UCNP‐COF, and ICG‐loaded UCNP‐COF (ICG@UCNP‐COF) particles. The ICG solution was prepared as an aqueous solution with a concentration of 500 µg mL^−1^, which was also used to load ICG into the UCNP‐COF by continuously stirring the UCNP‐COF particles in the ICG solution for 24 h. The bare ICG sample exhibited characteristic absorbance peaks at 714 and 778 nm.^[^ ^11^, ^12^, ^13^ ^]^ In contrast to the UCNP‐COF, the ICG@UCNP‐COF sample showed absorbance peaks ≈778 nm, indicating efficient loading of ICG into the UCNP‐COF particles. By comparing the 778 nm absorbance peak of the ICG solution before and after loading into UCNP‐COF particles, the ICG loading efficiency in the ICG@UCNP‐COF particles was calculated to be 91%.

Figure 3b shows the MSOT spectra of six different samples: CS‐UCNPs, TAPB‐TPA COF, ICG solution (ICG), UCNP‐COF, ICG‐loaded TAPB‐TPA COF (ICG@COF), and ICG@UCNP‐COF, all measured in tissue‐mimicking agar gel phantoms. The COF‐based particle samples (TAPB‐TPA COF, UCNP‐COF, ICG@COF, and ICG@UCNP‐COF) were dispersed within the agar gels at a concentration of 250 µg mL^−1^ and the agar gel mixtures were solidified inside the 2 mm‐diameter tubes. CS‐UCNPs and ICG samples were prepared as aqueous suspensions or solutions, respectively, with a concentration of 500 µg mL^−1^ and filled in the 2 mm‐diameter tubes. We focused on the wavelength range of 650 to 950 nm, as it can penetrate tissue without significant attenuation. Moreover, the contrast spectral signatures within this range do not overlap with the strongest absorption range by oxyhemoglobin and deoxyhemoglobin,^[^ ^1^, ^2^, ^3^ ^]^ enabling clear distinction of the contrast agents from the background absorption by blood.

Across the wavelength range of 950 to 650 nm, TAPB‐TPA COF showed no distinct MSOT spectral signature, while CS‐UCNPs exhibited weak but gradually increasing spectral profile. Both the ICG solution and ICG@COF displayed enhanced MSOT signals at ∼800 nm, with the ICG@COF sample producing signals ∼2.0 times stronger than those of the ICG solution. Similar to the enhanced absorbance peak of the ICG@UCNP‐COF shown in Figure 3a, this MSOT signal increase is attributed to the diffusion of ICG molecules followed by their encapsulation into the textural mesopores of the COF. As we discussed in our previous study,^[^ ^29^ ^]^ the molecular diameter of ICG along its longest axis (≈2.9 nm) allows it to diffuse into the 3.2 nm‐wide COF pores. Within these pores, ICG molecules intercalate through electrostatic and intermolecular interactions, significantly increasing the local concentration of ICG and thereby generating stronger MSOT signals than the bare ICG solution.

Notably, the UCNP‐COF sample exhibited substantially higher MSOT signal amplitude comparable to those of the ICG@COF. Its intensity was only ≈1.2 times lower than that of the ICG@COF sample and began to increase steeply at longer wavelengths compared to ICG@COF. When the ICG molecules were loaded into the UCNP‐COF particles, forming ICG@UCNP‐COF, it generated the highest signal among all the samples. At 800 nm, the ICG@UCNP‐COF produced a 1.6‐ and 1.9‐fold increase in MSOT signals compared to ICG@COF and UCNP‐COF, respectively, and displayed a similar tendency of signal increase to the UCNP‐COF. Additionally, we observed concentration‐dependent MSOT signal intensities at 808 nm, exhibiting proportional intensity enhancement with a fitted slope (*k*) of 12.9, 8.6, 7.3, and 2.6 for ICG@UCNP‐COF, ICG@COF, UCNP‐COF, and ICG sample, respectively (Figure 3c).

Figure 3d shows the temperature increase of the various samples tested in Figure 3b, subjected to continuous‐wave 808 nm NIR laser irradiation with a power density of 500 mW cm^−2^ over time. The temperature change was monitored using an infrared camera, as shown in the infrared image below the graph. The CS‐UCNPs and TAPB‐TPA COF samples exhibited minimal temperature changes under irradiation, reaching 28.9 °C and 31.0 °C, respectively, after 3 min. The ICG solution (500 µg mL^−1^ ICG concentration) showed a temperature rise to 46.3 °C, while the ICG@COF sample (200 µg mL^−1^ particle concentration) reached 68.5 °C during the same period. This enhanced heating effect in the ICG@COF is attributed to the encapsulation of a larger amount of ICG compared to the ICG solution, consistent with the MSOT data shown in Figure 3b. The UCNP‐COF sample exhibited a temperature increase to 64.8 °C after 3 min, supporting our hypothesis that UCL absorption by the COF may contribute to the thermoelastic expansion and MSOT signal generation of the particles. The ICG@UCNP‐COF sample demonstrated the most rapid and efficient heating, reaching 74.8 °C after 3 min.

From the MSOT and temperature measurements across the various samples in Figures 3b,d, we schematically depict the mechanism behind MSOT signal generation from the UCNP‐COF particles and its enhancement in the ICG@UCNP‐COF particles in Figure 3e. Under 808 nm NIR laser irradiation, Nd^3+^ ions in NaYF_4_:Yb,Tm@NaYF_4_@NaYF_4_:Yb,Nd@SiO_2_ CS‐UCNPs absorbed 808 nm photons and transferred the excitation energy to the energy bands of Tm^3+^ ions via Yb^3+^ ions through cascade energy transfer.^[^ ^20^, ^21^ ^]^ Consequently, Tm^3+^ emitter ions produced UCL emissions at 289 nm (^1^I_6_ → ^3^H_6_), 344 nm (^1^I_6_ → ^3^F_4_), 361 nm (^1^D_2_ → ^3^H_6_), 450 nm (^1^D_2_ → ^3^F_4_), 474 nm (^1^G_4_ → ^3^H_6_) and 649 nm (^1^G_4_ → ^3^F_4_). The NaYF_4_ shell layer between NaYF_4_:Yb,Tm core, and NaYF_4_:Yb,Nd shell layers acts as an energy barrier to avoid the energy back‐transfer from Yb^3+^ to Nd^3+^ bands,^[^ ^21^ ^]^ enhancing luminescence intensities.

In UCNP‐COF, where the TAPB‐TPA COF with an E_g_ of 2.6 eV encapsulates CS‐UCNPs, photons from the CS‐UCNPs with wavelengths below 477 nm are absorbed by the surrounding COF, quenching the UCL emission. This quenching process converts the energy that would have been emitted as photons into heat and vibration (thermal expansion) within the COF, leading to the generation of acoustic waves and, consequently, MSOT signal detection. Similar to our findings, previous studies have demonstrated that mixing or conjugating nitro dyes containing azobenzene^[^ ^26^ ^]^ or nitrile groups,^[^ ^27^ ^]^—which absorb light in the UV‐blue wavelength range—with Tm^3+^‐doped UCNPs can generate MSOT signals through UCL quenching. Therefore, we propose that the primary mechanism of MSOT signal generation in our UCNP‐COF particles is luminescence quenching, consistent with previous observations.^[^ ^25^, ^26^, ^27^ ^]^ However, unlike previous studies, the highly ordered porous framework of the UCNP‐COF particles provides the additional advantage of loading desired molecules, such as ICG, to further increase NIR absorption, thereby enhancing the MSOT signal intensity beyond what can be achieved with UCNP‐COF alone.

Deep‐tissue imaging is crucial for precise diagnosis, and MSOT imaging combined with well‐designed contrast agents can offer significant penetration depth and high spatial resolution.^[^ ^1^, ^2^, ^3^, ^4^, ^5^ ^]^ MSOT is known to achieve penetration depths up to several centimeters, with imaging resolutions down to 100–200 µm.^[^ ^1^, ^2^ ^]^ Furthermore, long‐term stability is essential for contrast agents used in the continuous monitoring of diseases or conditions over extended periods, ensuring consistent image quality. To test the MSOT imaging penetration depth, we placed different samples prepared within agar‐containing tubes at various depths (*d*). Agar gel was poured over the tube samples and allowed to solidify, forming an agar phantom with a thickness of either 10 or 20 mm. Figure 3f and Figure S7 (Supporting Information) compare the relative MSOT signal intensities of the ICG@UCNP‐COF, UCNP‐COF, and ICG solution samples at different depths within the agar phantom. All samples were prepared at the same concentration as those tested in Figure 3b. At *d* = 10 mm, both ICG@UCNP‐COF and UCNP‐COF samples exhibited strong, comparable MSOT signals. However, at *d* = 20 mm, the signal from the UCNP‐COF sample was slightly weaker but still comparable to that of the ICG@UCNP‐COF. The MSOT signals from the ICG solution were relatively lower in intensity compared to the other samples at both depths. Additionally, we evaluated the long‐term stability of the UCNP‐COF, ICG@COF, and ICG solution samples in water over 30 days. After aging in deionized water for one month under room light, the UCNP‐COF particles maintained their initial contrast performance, whereas the ICG‐containing samples (ICG@COF and ICG solution) showed a substantial decrease in signal intensity, demonstrating the environmental instability of ICG.

To demonstrate the effectiveness of UCNP‐COF particles for deep‐penetrating in vivo MSOT imaging, we conducted non‐invasive volumetric imaging of the brain vasculature in live mice, keeping both the scalp and skull intact (Figure 3g and Movie S1, Supporting Information). The dense and irregular structures of the skin, skull, and underlying tissues not only reduce the amount of NIR light reaching the cerebral vessels but also attenuate and scatter acoustic waves. During imaging, we injected a certain amount (200 µL, 500 µg mL^−1^) of UCNP‐COF particles into the tail vein through a bolus injection. Before the injection, only the major superior sagittal sinus (SSS) was visible in the *xy*‐view images, with the surrounding vessels barely distinguishable. However, two seconds after injection, anterior cerebral artery (ACA), and superior anastomotic vein, other branched cortical veins connected to the SSS became visible with substantially enhanced signal intensities, indicating the successful circulation of the UCNP‐COF particles within the cerebral vasculature. Even 12 seconds post‐injection, the cerebral vessels remained observable with higher signal intensities compared to the pre‐injection state. The *yz*‐ and *xz*‐views were also obtained, along with the *xy*‐view (Movie S1, Supporting Information), thus generating 3D MSOT views that can potentially track contrast agents through real‐time monitoring.

Furthermore, we evaluated the long‐term stability of the UCNP‐COF particles in vivo by storing them in water for 4 months in the dark. To assess their in vivo MSOT signal generation, we injected the particles into the femoral vasculature of mice (Figure S8, Supporting Information). Despite prolonged exposure to water, the 4‐month‐old UCNP‐COF particles produced enhanced MSOT contrast within 1 min of injection. The signal intensity observed in the femoral vessel gradually declined over 20 min, indicating a body circulation time of the UCNP‐COF particles <20 min.

### 2.3 Improved Biocompatibility and Drug Loading & Release Capability

**Figure 4:**
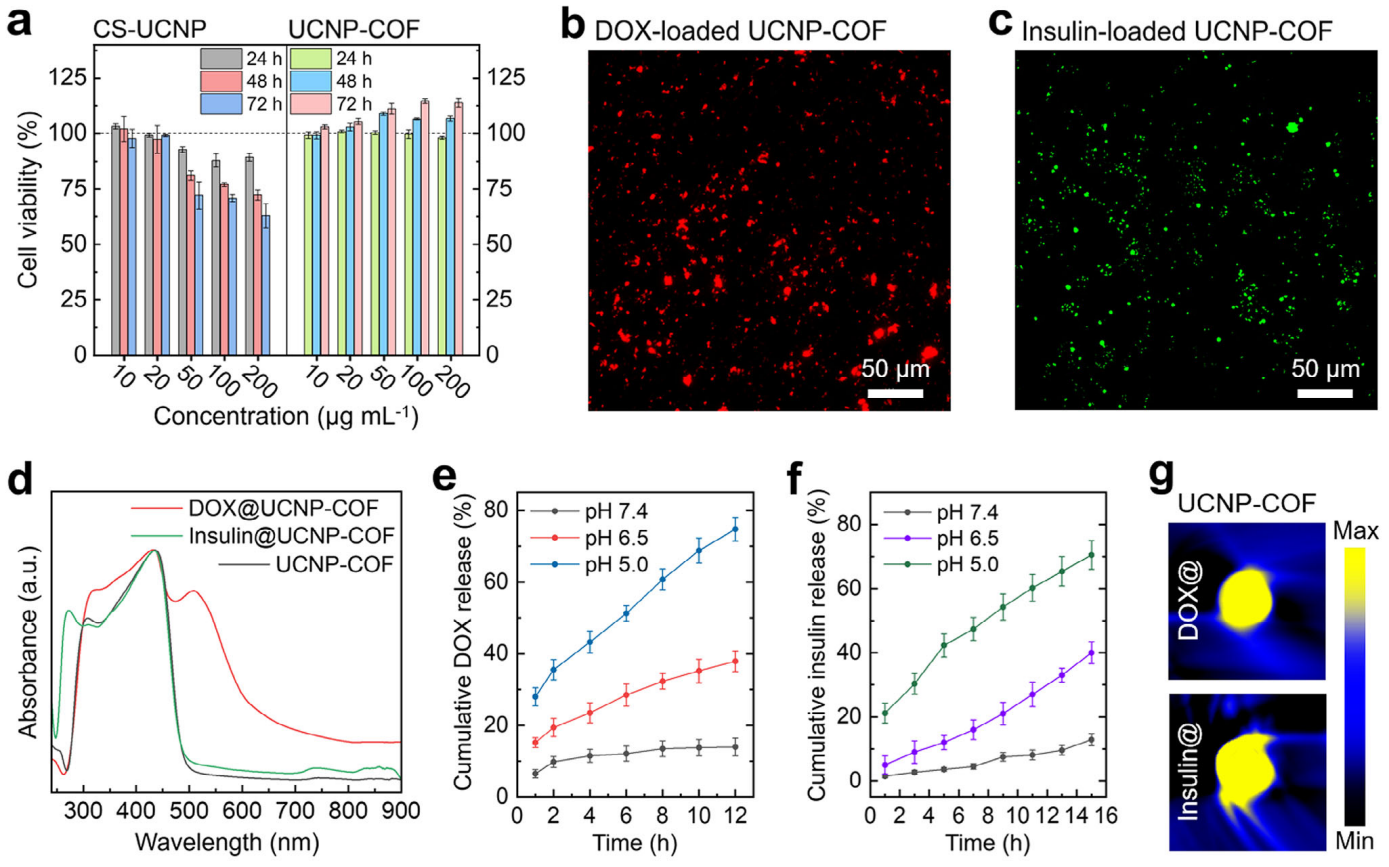
Biocompatibility assessment and therapeutic agent loading/release characterization. a) Viability of fibroblast cells exposed to various concentrations of CS‐UCNPs and UCNP‐COF particles over 24, 48, and 72 h. b,c) Fluorescence microscopy images of DOX (red)‐loaded (b), and FITC‐labeled insulin (green)‐loaded (c) UCNP‐COF particles. d) Absorption spectra of UCNP‐COF, DOX@UCNP‐COF, and insulin@UCNP‐COF particles. e,f) Cumulative DOX (e) and insulin (f) release from UCNP‐COF particles under different pH conditions. g) MSOT imaging of DOX@‐ and insulin@UCNP‐COF particles embedded within an agar phantom.

For potential biomedical applications, UCNP‐COF particles should exhibit minimal cytotoxicity. To evaluate this, we tested the cytotoxicity of CS‐UCNPs and UCNP‐COF particles using cultured human skin fibroblast cells (**Figure** **4**a). Fibroblast cells were incubated with varying concentrations (10−200 µg mL^−1^) of CS‐UCNPs and UCNP‐COF particles in the dark, and cell viability was monitored over 72 h. The CS‐UCNPs demonstrated a reduction in cell viability at concentrations above 50 µg mL^−1^, with viability dropping to ≈60% after 72 h of incubation. This cytotoxicity is likely due to the presence of lanthanide materials and silica layers in the CS‐UCNPs, which are known to induce toxicity in cells.^[^ ^17^, ^18^ ^]^ In contrast, UCNP‐COF particles exhibited no cytotoxicity to the fibroblast cells across all tested concentrations and incubation periods. Remarkably, after 72 h of incubation at higher concentrations (100 and 200 µg mL^−1^), cell viability increased to as much as 110% with UCNP‐COF particles. This improvement in cell viability, compared to CS‐UCNPs, is attributed to the encapsulation of the CS‐UCNPs with TAPB‐TPA COF layers, composed solely of nitrogen and carbon atoms, which significantly enhances their biocompatibility, as well as to their mesoporous nanostructures which likely enhance cell proliferation by facilitating cell adhesion, spreading, and growth.

To assess the potential of UCNP‐COF particles for therapeutic drug delivery while also functioning as contrast agents for MSOT imaging, we evaluated their capacity to carry different pharmacological agents. Two therapeutic agents were selected: doxorubicin (DOX), a chemotherapeutic drug widely used to treat various cancers, and insulin, a peptide drug commonly used for diabetes management. The molecular diameters of DOX and insulin are ≈1.1 and 2.7 nm, respectively,^[^ ^29^, ^30^ ^]^ which makes them suitable for efficient adsorption into the inner structural (3.2 nm pore width) and textural pores of the UCNP‐COF particles. The positive zeta potentials (ζ) of the TAPB‐TPA COF (ζ = 12.1 ± 1.3 mV) and UCNP‐COF (ζ = 10.8 ± 2.5 mV) particles resulting from the protonation of their amine groups, along with the substantial dipole moments of their imine groups, help reduce agglomeration and enhance drug adsorption efficiency.^[^ ^29^, ^31^ ^]^ After 24 h of stirring the red fluorescent DOX (595 nm emission) or fluorescein isothiocyanate (FITC)‐labeled insulin (517 nm emission) in a phosphate‐buffered saline (PBS) solution (200 µg mL^−1^ concentration) with UCNP‐COF particles (500 µg mL^−1^ concentration), successful drug loading was confirmed through fluorescence microscopy (Figures 4b,c) and UV–Vis spectroscopy (Figure 4d). Based on the changes in absorbance intensity of the DOX‐ or insulin‐dissolved PBS solution before and after loading into UCNP‐COF particles, the loading efficiencies were determined to be 94% for DOX and 89% for insulin.

The therapeutic agents loaded into the UCNP‐COF particles should be released at targeted locations via specific triggers. It is well established that a shift to acidic conditions facilitates the release of DOX or insulin from nano‐ or mesoporous structures at the target site.^[^ ^29^, ^30^, ^31^, ^32^ ^]^ In our study, we adjusted the environmental pH of the DOX‐loaded UCNP‐COF (DOX@UCNP‐COF) particles from physiological pH (pH 7.4) to a lower pH (pH 5.0) by adding acetic acid to PBS. We then quantified the amount of DOX released over 12 h and compared it to the initially loaded amount in the UCNP‐COF particles (Figure 4e). After 12 h at pH = 7.4, only 14% of DOX was released, which implies that the DOX adsorbed on the particle surface of the UCNP‐COF particles was diffused out, while the DOX molecules encapsulated within the structural mesopores remained stably loaded. Reducing the pH to 6.5 and 5.0 triggered a DOX release of 38% and 75%, respectively, after 12 h. The low pH promotes the hydrolysis of the acid‐cleavable hydrazone linkages of the DOX molecules,^[^ ^32^ ^]^ destabilizing the loaded DOX and facilitating its release from the mesopores of the UCNP‐COF particles into the surrounding environment. Tumor tissues, inflamed tissues, and intracellular compartments such as endosomes and lysosomes typically have a more acidic environment than normal tissues.^[^ ^33^ ^]^ This lower pH can potentially enable targeted therapy in tumor or infection sites, which generally exhibit more acidic conditions. Similar to the release of DOX from UCNP‐COF particles, insulin release was also mediated by pH control. After incubating insulin‐loaded UCNP‐COF (Insulin@UCNP‐COF) particles at pH 7.4 for 15 h, only 12% of the preloaded insulin was released. However, when the pH was decreased to 6.5 and 5.0, the released insulin increased to 40% and 71%, after the same incubation period (Figure 4f). This insulin release triggered by acidic conditions can occur in the body through gluconic acid, which is a catalytic product of glucose.^[^ ^34^ ^]^

Lastly, we performed MSOT imaging of the DOX@UCNP‐COF and insulin@UCNP‐COF particles under 808 nm illumination wavelength (Figure 4g). Both samples were prepared using the same method as in Figure 3f and were placed within an agar gel phantom located at *d* = 10 mm. The MSOT signals generated by both DOX‐ and insulin‐loaded samples were substantial and comparable to those from the UCNP‐COF particles without therapeutic agent loading. This indicates that the presence of loaded DOX or insulin does not affect the MSOT signal generation mechanism, which relies on UCL quenching at the interface between CS‐UCNPs and the TAPB‐TPA COF.

### 2.4 In Vivo MSOT Imaging and Magnetic Steering of UCNP‐COF Particles

To wirelessly steer imaging contrast agents while monitoring via real‐time imaging, we transformed UCNP‐COF particles into magnetic Janus particles by sputtering Ni─Au layer on the surface of the particles, which have been widely used to fabricate magnetic microrobots or microrollers.^[^ ^19^, ^35^ ^]^ First, we created a monolayer of UCNP‐COF particles by drop‐casting an aqueous solution of UCNP‐COF particles onto an O_2_ plasma‐treated glass substrate, followed by slow water evaporation over 12 h. Then, magnetic Janus particles were fabricated by sequentially sputtering 50 nm‐Ni and 20 nm‐Au on the prepared layer of particles using a sputter coating system, resulting in a Janus‐type particle. After coating, magnetization was conducted to the out‐of‐plane direction of the substrate under uniform magnetic fields. Lastly, the sample was placed in an ethanol bath and the sonication‐induced Janus particles were detached from the substrate in a few seconds. The as‐fabricated Janus particles were collected by evaporating ethanol. The fabrication process is depicted in Figure S9 (Supporting Information).

**Figure 5:**
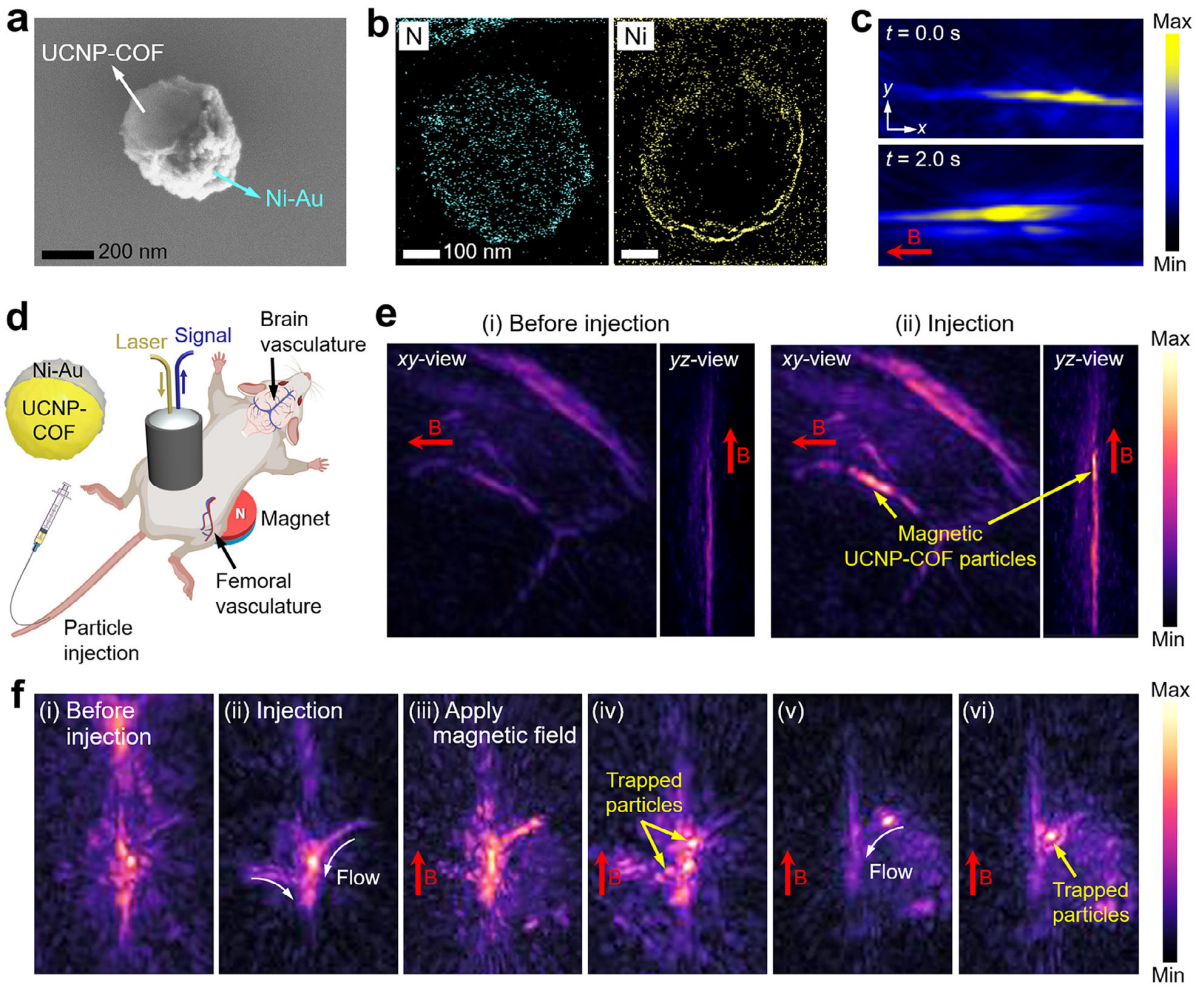
In vivo real‐time 3D MSOT imaging and magnetic steering of magnetic Janus UCNP‐COF particles. a) SEM image and b) EELS elemental mapping b) of a Janus Ni─Au‐sputtered UCNP‐COF particle. c) MSOT imaging of the motion of magnetic Janus particles under a magnetic field. d) Schematic illustration of in vivo real‐time 3D MSOT imaging and magnetic steering of Janus particles within femoral and brain vasculature of a mouse. e) In vivo MSOT imaging of femoral vasculature i) before and ii) after particle injection, shown in both *xy*‐ and *yz*‐views. f) In vivo MSOT imaging of brain vasculature before and after particle injection, with particles trapped under a magnetic field.

**Figure** **5**a,b shows SEM and electron energy loss spectroscopy (EELS) images of the Janus particles, where one‐half of the UCNP‐COF particle is sputtered with a Ni─Au layer. The Ni─Au sputtered Janus particles exhibited a similar average diameter of 430 nm to that of the non‐coated UCNP‐COF particles. Compared to previously studied magnetically driven microparticles or microrobots, which range in size from several micrometers^[^ ^19^, ^35^ ^]^ to hundreds of micrometers,^[^ ^36^ ^]^ the nanometer‐sized Janus particles are expected to be optimal for the enhanced permeability and retention (EPR) effect.^[^ ^37^ ^]^ Their sizes allow them to pass through tumor vasculature pores, typically ranging from 100 to 780 nm,^[^ ^37^ ^]^ or to be cleared by macrophages through endocytosis and phagocytosis.^[^ ^38^ ^]^ The Janus particles exhibited a surface area of 525.4 m^2^ g^−1^, which is lower than that of the UCNP‐COF particles (654.9 m^2^ g^−1^) (Figure S10, Supporting Information). This reduction could be attributed to the Ni─Au layer on the particles. However, the particles still exhibit a relatively high surface area compared to other COF‐based studies.^[^ ^29^, ^31^ ^]^ Sputtering with a Ni─Au layer enabled precise control over the thickness of the magnetic Ni layer on the UCNP‐COF particles. When a relatively thick Ni layer (150 nm) was applied, the Janus particles tended to agglomerate due to their strong magnetic interactions (Figure S11, Supporting Information). However, with a thinner Ni (50 nm)‐Au magnetic nanofilm, the Janus particles could be steered by a neodymium (NdFeB) magnet while remaining dispersed in the fluid, enabling collective control of their movement without particle agglomeration. Under a directional magnetic field, the magnetic Janus particles in water separately moved with an average velocity of 6.53 ± 0.04 µm s^−1^, as confirmed by optical microscopy tracking (Figure S12, Supporting Information), and also observed through MSOT imaging (Figure 5c).

Figure 5d schematically illustrates the experimental setup for in vivo real‐time 3D MSOT imaging and magnetic steering of the Ni─Au‐sputtered Janus UCNP‐COF particles within the femoral and brain vasculature of two mice. First, the MSOT probe was positioned on the hindlimb of a mouse, with ultrasound gel applied, allowing for real‐time 3D imaging of the femoral vessels to monitor the movement of particles within the bloodstream. It has been studied that the blood flow velocity within the mice's femoral vein corresponds to 16 mm s^−1^.^[^ ^39^ ^]^ A magnet was placed beneath the mouse to steer the particles toward the torso. When the magnetic Janus particles dispersed in 30 µL PBS with a concentration of 500 µg mL^−1^ were injected into the mouse tail vein, we observed substantial MSOTcontrast signal increases within the femoral vessels, as well as the magnetic steering of particles to the direction of the magnetic field (Figure 5e; Movie S2, Supporting Information).

We further tested Janus UCNP‐COF particles within the brain vasculature of another mouse (Figure 5f; Movie S3, Supporting Information). Cerebral blood flow in brain vessels, such as the SSS, has been reported to reach velocities of over 50 mm s^−1^,^[^ ^40^ ^]^ which is approximately three times higher than the velocity in femoral veins. Comparing the i) MSOT image before particle injection, (ii) to the post‐injection image, we observed an increase in contrast signals and a clear flow of the magnetic UCNP‐COF particles immediately following tail vein injection. iii) When a magnetic field was applied by placing a permanent magnet beneath the mouse's head, iv) we observed a further increase in MSOT signal intensity, along with the trapping of the particles near the magnet. This effect seems to be due to the particles being attracted toward the vessel walls close to the magnet. v) Although particles were only temporarily trapped due to the significantly high cerebral blood flow, we could continuously monitor the steering and vi) subsequent trapping of the particles under the magnetic field.

Despite the substantial biocompatibility of UCNP‐COF particles, concerns regarding their long‐term effects on metabolism and circulatory function should be addressed. To investigate this, we performed two‐photon microscopy imaging on sliced post‐mortem liver and brain to track the histological distribution and accumulation of particles (Figure S13, Supporting Information). Since both the liver and brain, particularly the liver, exhibit autofluorescence due to intrinsic fluorophores such as nicotinamide adenine dinucleotide (NAD),^[^ ^41^ ^]^ we injected ICG@UCNP‐COF particles, which exhibit strong optical absorption at 780 nm excitation, to differentiate particles from surrounding tissues. The excitation wavelength was alternated between 780 and 900 nm, as ICG@UCNP‐COF particles have relatively lower absorption at 900 nm compared to 780 nm.

According to the literature, particles of ≈400 nm in size are primarily cleared by the liver.^[^ ^42^ ^]^ As expected, liver tissues from mice injected with particles and excited at 780 nm exhibited fluorescence signals corresponding to ICG@UCNP‐COF particles, while these signals were negligible at 900 nm. In contrast, brain tissues from injected mice showed no fluorescence difference between 780 and 900 nm excitation, similar to the liver samples from non‐injected mice. These results demonstrate that UCNP‐COF particles accumulate in the liver and are effectively cleared, while they do not cross the blood‐brain barrier (BBB) or accumulate in the brain.

### 2.5 Targeted Drug Release by MSOT‐Trackable Microrobots

**Figure 6:**
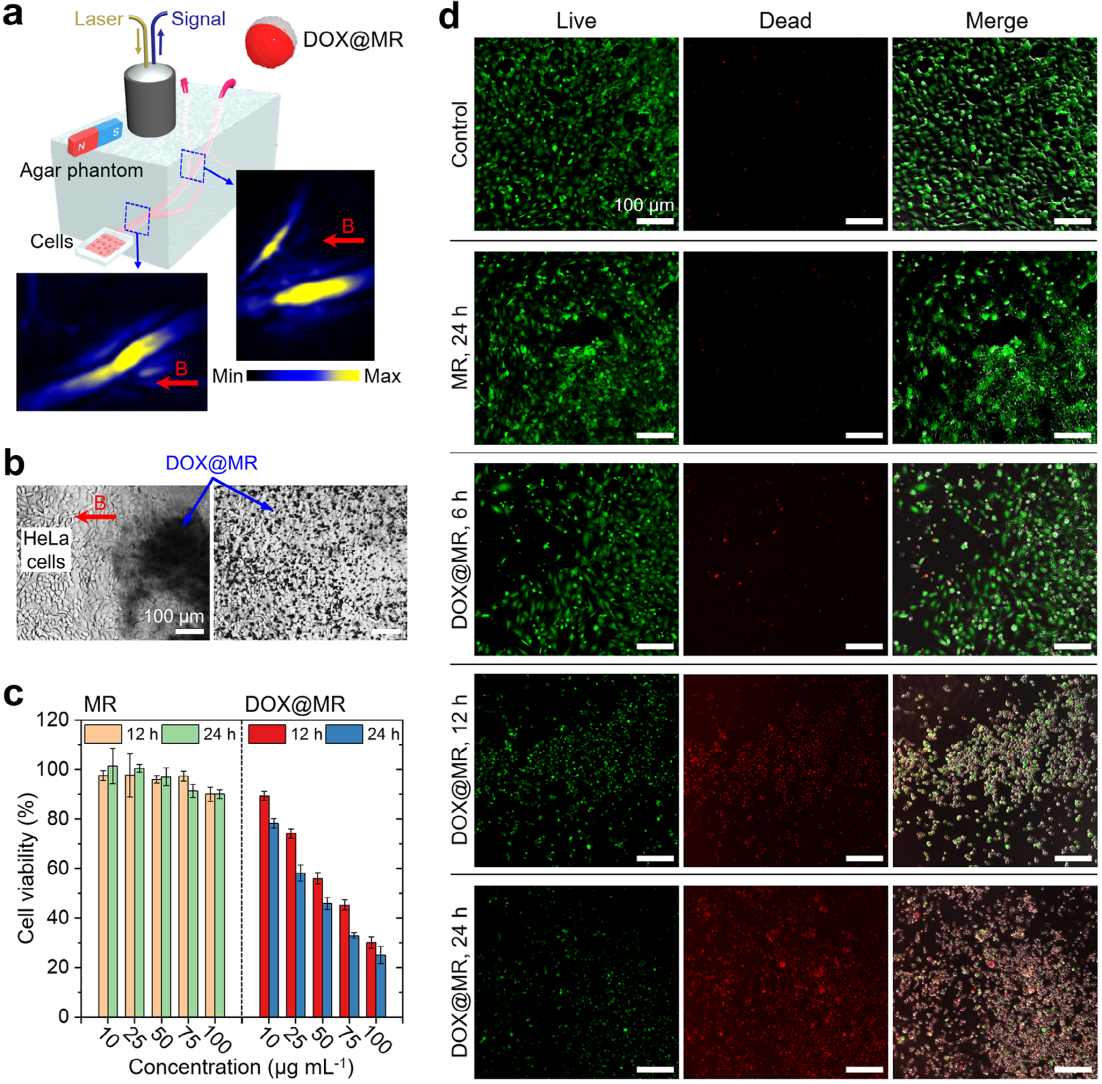
Therapeutic capability of MSOT‐trackable microrobots. a) Schematic setup for magnetic steering of DOX‐loaded microrobots (DOX@MRs) through the in vitro vascular channels and MSOT imaging of the motion of DOX@MRs guided toward HeLa cells. b) Optical microscope images showing the arrival and distribution of DOX@MRs on the HeLa cells. c,d) Concentration‐, and time‐dependent cytotoxicity of MRs without DOX and DOX@MRs against HeLa cells, assessed by cell viability (c) and fluorescent live/dead microscopy assay (d).

Microrobots with wireless controllability, medical imaging tracking, and therapeutic capabilities have gained substantial attention.^[^ ^43^ ^]^ In **Figure** **6**, we evaluated the Janus UCNP‐COF particles as MSOT‐trackable therapeutic microrobots (MRs). For this, in vitro artificial 3D vascular channels were created within an agar phantom, and an external magnetic field was used to steer DOX‐loaded MRs (DOX@MRs) to the end of the channel, where HeLa cells—a well‐studied cancer cell line^[^ ^44^ ^]^—were seeded. The DOX@MRs were prepared similarly to the DOX@UCNP‐COF particles shown in Figure 4, except that Ni─Au‐sputtered Janus UCNP‐COF particles were used. The vascular channels were filled with a static Dulbecco's modified Eagle medium (DMEM), and 100 µg mL^−1^ of DOX@MRs were injected at the channel's end. During magnetic guidance of the DOX@MRs, the MSOT imaging probe positioned above the agar phantom tracked the DOX@MRs. Clear signals detected by the probe visualized the motion of DOX@MRs as they converged and progressed from two separate channels into a single channel (Figure 6a).

Using magnetic steering assisted by MSOT imaging, the DOX@MRs successfully navigated through the in vitro channels and reached the HeLa cells, as confirmed by optical microscopy (Figure 6b). We then examined the interaction between the DOX@MRs and HeLa cells using a 3‐(4,5,‐dime‐thylthiazol‐2‐yl)‐2,5‐diphenyltetrazolium bromide (MTT) cytotoxicity assay (Figure 6c) and a confocal fluorescence live/dead assay (Figure 6d). For comparison, we assessed a control group of untreated HeLa cells, HeLa cells exposed to MRs without DOX (referred to as MRs), and groups exposed to various concentrations of either MRs and DOX@MRs (10, 25, 50, and 75 µg mL^−1^). In both the MTT and fluorescence assays, MRs without DOX showed no impact on HeLa cells after 24 h, regardless of concentration. In contrast, HeLa cells incubated with DOX@MRs for 6, 12, and 24 h demonstrated a gradual increase in cytotoxicity due to the release of DOX from the MRs, as confirmed by a decrease in cell viability with higher DOX@MRs concentrations and longer exposure times. Additionally, we investigated the targeted magnetic steering of the DOX@MRs to 3D HeLa tumor spheroids and assessed spheroid viability following DOX release from the MRs (Figure S14, Supporting Information). Using magnetic field‐assisted steering, the DOX@MRs were successfully localized to the 3D spheroid, which was then incubated for 3 days while assessing spheroid viability daily. The spheroid viability progressively declined, with values of 92%, 56%, 45%, and 32% on days 0, 1, 2, and 3, respectively.

The magnetic steering capability of the injected Janus UCNP‐COF therapeutic MRs enabled their magnetic retrieval from the body, potentially mitigating side effects. Using a catheter with a magnetic tip attached (Figure S15a, Supporting Information), we demonstrated the retrieval of magnetic UCNP‐COF particles from phantom blood vessels while visualizing the process through 3D MSOT imaging (Figure S15b, Supporting Information). Before retrieval, the particles were distributed within separate bifurcated vessels. As the magnetic tip approached the vessel end, the particles converged into a single cluster due to the strong magnetic attraction and were subsequently retrieved upon attachment to the magnetic tip. This approach enables magnetic targeted therapy combined with efficient elimination via a magnetic field, with real‐time monitoring in 3D through MSOT imaging.

## 3 Conclusion

In this study, we introduce UCNP‐COF particles as multifunctional agents capable of achieving simultaneous MSOT contrast generation, therapeutic delivery, and microrobotic capabilities within a single platform. These UCNP‐COF particles, trackable via MSOT imaging in 3D and real‐time, can concurrently load and release therapeutic agents while being magnetically guided through biological environments. The particles generate MSOT signals by quenching the UV and blue UCL emitted by UCNPs, which the encapsulating COF absorbs and converts into detectable ultrasound waves. Compared to traditional MSOT imaging contrast agents, UCNP‐COF particles offer superior imaging depth, long‐term environmental stability, and biocompatibility. The highly ordered, mesoporous COF matrix provides a large surface area for customizable therapeutic loading and controlled release at target sites. Additionally, magnetic Janus transformation enables magnetic navigation of these UCNP‐COF particles through in vivo vasculature, allowing for real‐time volumetric tracking via MSOT. Our findings highlight a novel design strategy for MSOT imaging contrast agents, leveraging the interaction between luminescent and absorptive properties of different photonic nanomaterials,^[^ ^45^, ^46^ ^]^ while integrating drug delivery, and microrobotic functionality into a single, streamlined system.

## 4 Experimental Section

### Synthesis of Core NaYF_4_:Yb,Tm@NaYF_4_@NaYF_4_:Yb,Nd UCNPs

The core UCNPs with a composition of NaYF_4_:Yb,Tm@NaYF_4_@NaYF_4_:Yb,Nd were synthesized by first preparing NaYF_4_:Yb,Tm UCNPs, followed by the sequential formation of NaYF_4_ and NaYF_4_:Yb,Nd layers. First, for the synthesis of NaYF_4_:Yb,Tm UCNPs, 226.6 mg of yttrium(III) chloride hexahydrate (YCl_3_·6H_2_O, 99.99%, Sigma–Aldrich), 96.9 mg of ytterbium(III) chloride hexahydrate (YbCl_3_·6H_2_O, 99.99%, Sigma–Aldrich), and 1.15 mg of thulium(III) chloride hexahydrate (TmCl_3_·6H_2_O, 99.99%, Sigma–Aldrich) were added to a 50 mL three‐neck flask. Then, 15 mL of 1‐octadecene (ODE, Sigma–Aldrich) and 6 mL of oleic acid (OLA, Sigma–Aldrich) were added, and the mixture was heated to 150 °C for 30 min with magnetic stirring at 350 rpm to obtain a homogeneous, transparent solution. After cooling to 60 °C, 0.148 g of ammonium fluoride (NH_4_F, 99.99%) and 0.1 g of sodium hydroxide (NaOH, 98%, Sigma–Aldrich) were dissolved in 5 mL of methanol (99.8%, Carl Roth) by 30 min sonication and added to the solution dropwise. The reaction mixture was heated to 120 °C for 20 min to fully evaporate methanol and residual moisture. After the evaporation of methanol, the neck of the flask was blocked with a rubber stopper and connected to the dual manifold line through a condenser. The solution was kept in a vacuum for 10 min and filled with nitrogen. The solution was heated to 300 °C and the temperature was maintained under nitrogen for 1 h with stirring (350 rpm). The mixture was cooled to room temperature naturally and transferred to a 50 mL conical tube. The tube containing the solution was centrifuged at 6654 *g* for 10 min. After removing the supernatant, the precipitate was dispersed in 10 mL of cyclohexane (anhydrous, Sigma–Aldrich). The solution was centrifuged again at 1000*g* for 5 min using cyclohexane, and the supernatant containing NaYF_4_:Yb,Tm UCNPs were collected.

To form a NaYF_4_ layer on the pre‐synthesized NaYF_4_:Yb,Tm UCNPs, 242.7 mg of YCl_3_·6H_2_O was added to a 50 mL three‐neck flask and then mixed with 15 mL of ODE and 6 mL of OLA. The mixture solution was heated to 150 °C and stirred at 350 rpm for 30 min to obtain a homogeneous, transparent solution. After cooling to 60 °C, pre‐synthesized NaYF_4_:Yb,Tm UCNPs dispersed in 5 mL of cyclohexane were added, followed by 5 mL of methanol solution containing NH_4_F (0.148 g) and NaOH (0.1 g). The mixture was stirred for 30 min at 60 °C. After this period, the reaction mixture was heated to 120 °C for 20 min to fully evaporate methanol, cyclohexane, and any residual moisture. Once solvent evaporation was completed, a rubber stopper blocked the flask neck, which was then connected to a dual manifold line through a condenser. The solution was held under a vacuum for 10 min, and then filled with nitrogen. Subsequently, the temperature was raised to 300 °C and maintained under nitrogen with stirring at 350 rpm for 1 h. The mixture was then allowed to cool naturally to room temperature and transferred to a 50 mL conical tube. The solution was centrifuged at 6654*g* for 10 min, and the supernatant was removed. The precipitate was re‐dispersed in 5 mL of cyclohexane and centrifuged again at 1000*g* for 5 min. Finally, the supernatant containing NaYF_4_:Yb,Tm@NaYF_4_ UCNPs were collected. To synthesize the outermost layer of NaYF_4_:Yb,Nd, the previously described process was repeated using YCl_3_·6H_2_O (129.4 mg), NdCl_3_·6H_2_O (161.8 mg, 99.9%, Sigma–Aldrich), and YbCl_3_·6H_2_O (32.3 mg) as initial precursors, along with the addition of NaYF_4_:Yb,Tm@NaYF_4_ UCNPs during the synthesis.

### Synthesis of CS‐UCNPs

Five milliliters of as‐synthesized NaYF_4_:Yb,Tm@NaYF_4_@NaYF_4_:Yb,Nd UCNPs dispersed in cyclohexane was added dropwise to a solution of 3 mmol IGEPAL CO‐520 (average M_n_ of 441, Sigma–Aldrich) dissolved in 12 mL cyclohexane under stirring (500 rpm). Subsequently, 160 µL of tetraethyl orthosilicate (TEOS, Sigma–Aldrich) was injected into the mixture while stirring. After 15 min, 150 µL of ammonium hydroxide solution (28−30 wt.% in H_2_O, Sigma–Aldrich) was added. The resulting mixture was stirred in the dark for 24 h to facilitate SiO_2_ layer growth. After this period, 3 mL of ethanol was added to the solution, followed by centrifugation at 10 000 g. The supernatant was discarded, and the sediment was re‐dispersed in 10 mL ethanol (99.8%, Carl Roth). The dispersion was centrifuged again (10 000 g), and the sediment was washed twice more with ethanol before being re‐dispersed in acetonitrile (99.5%, Carl Roth), resulting in a slightly turbid solution of CS‐UCNPs.

### Synthesis of UCNP‐COF and TAPB‐TPA COF Particles

The synthesis of the UCNP‐COF colloidal particles was adapted from the reported procedure for TAPB‐TPA COF synthesis.^[^ ^47^ ^]^ Initially, 5.96 mg of terephthalaldehyde (TPA, 99.0%, Sigma–Aldrich) and 10.4 mg of 1,3,5‐tris(4‐aminophenyl)benzene (TAPB, > 93.0%, TCI Chemicals) were dissolved in 3 mL of acetonitrile. Subsequently, 150 µL of CS‐UCNP dispersion (50 mg mL^−1^ in acetonitrile) was added to this solution and sonicated for 10 min. While stirring gently with a magnetic stir bar (100 rpm), a solution of 3.5 mg scandium (III) trifluoromethane sulfonate (Sc(OTf)_3_, 99.0%, Sigma–Aldrich) in 3.8 mL acetonitrile was added dropwise over 10 min. The reaction proceeded for 24 h at room temperature. Following this, the solvent was exchanged with deionized water by centrifugation five times at 795 g for 10 min each cycle. The pristine TAPB‐TPA COF particles were synthesized using the same procedure but without the addition of CS‐UCNPs. For particle characterization, the synthesized UCNP‐COF and TAPB‐TPA COF particles were precipitated by adding 0.5 mL of 1 m sodium chloride (NaCl, 99.8%, Carl Roth) solution, washed with methanol, and dried using supercritical CO_2_ on a Leica EM CPD300 instrument.

### Characterizations

Scanning electron microscopy (SEM) images were obtained using a Gemini Ultra 55 (Carl Zeiss). High‐resolution transmission electron microscopy (HR‐TEM) images were acquired using a CM30 ST instrument (Philips) operating at 300 kV with a LaB_6_ cathode. HR‐TEM samples were carefully dried onto copper lacey carbon grids from Plano. Electron energy loss spectroscopy (EELS) elemental mapping was conducted using a JEM‐2200FS TEM instrument (JEOL) at the National Institute for Nanomaterials Technology (NINT), Republic of Korea. N_2_ adsorption/desorption isotherms were measured using a Quantachrome Autosorb iQ 3 at 77 K. The pore size distribution (PSD) was calculated based on N_2_ adsorption at 77 K on carbon, using the QSDFT model (cylindrical pores, adsorption branch) in ASiQwin software v3.01. Before measurement, samples were activated under a high vacuum at 120 °C for 12 h. For surface area determination, an optimal pressure range (*P*/*P*_0_ = 0.05−0.2) was selected. Powder X‐ray Diffraction (XRD) patterns were collected at room temperature using a Stoe Stadi P diffractometer (Cu‐Kα1) equipped with a Ge (111) primary monochromator, following the Debye‐Scherrer geometry. Each sample was securely sealed within 1.0 mm glass capillaries, and the measurements were taken with rotation to enhance particle statistics. Fourier‐transform infrared (FT‐IR) measurement was performed in attenuated total reflection (ATR) mode using a Spectrum Two FT‐IR spectrometer (PerkinElmer). UV–Vis spectra were obtained using a Cary 5000 UV–Vis–NIR spectrophotometer (Agilent) equipped with an integration sphere in absorptance mode. Fluorescence spectra of the samples were obtained using an 808 nm near‐infrared (NIR) laser (1700 mW, ZQ1 laser, Edmund Optics) operating in continuous‐wave mode. The emission spectra from the sample were acquired using a charge‐coupled device (CCS100, Thorlabs) and spectrofluorometer (Fluoromax, Horiba). Decay profile and lifetimes were measured using a time‐correlated single‐photon counting (TCSPC) system (MultiHarp 150, PicoQuant) with a 448/20 bandpass. Temperature changes in the samples were measured using an infrared thermal imaging camera (ETS320, FLIR). The Zeta potential of the particle samples was determined using a Zetasizer Nano Zs (Malvern Instruments).

### Multispectral Optoacoustic Tomography (MSOT) Imaging

MSOT measurements in phantoms were conducted using the inVision 512‐echo imaging system (iThera Medical GmbH, Munich, Germany). To acquire optoacoustic signals from various samples, powder samples were dispersed in an agar aqueous gel (agar concentration of 1.4 wt.%). The agar‐powder mixtures were then injected into cylindrical tubes (2 mm diameter) and allowed to solidify at room temperature. In parallel, CS‐UCNP and ICG solutions were directly injected into the tubes. Each cylindrical tube, filled with a specific sample, was subsequently inserted into an agar phantom with preformed cylindrical voids. MSOT spectra were obtained by scanning the samples across a wavelength range of 950 to 650 nm.

### Cell Cytotoxicity Test

Human skin fibroblast cells (BJ, American Type Culture Collection) were cultured in high‐glucose Dulbecco's modified Eagle medium (DMEM, Gibco), supplemented with 10% fetal bovine serum (FBS, Gibco) and 1% penicillin/streptomycin (Gibco), in 75 cm^2^ polystyrene cell culture flasks at 37 °C and 5% CO_2_. Once the cells reached 80% confluency, they were detached using a 0.25% trypsin‐EDTA solution (Gibco). The fibroblast cells were then seeded in a 96‐well plate with a black/clear bottom (Corning) at a density of 1 × 10^4^ cells per well, allowing them to adhere overnight. The following day, the culture media were replaced with DMEM containing CS‐UCNPs and UCNP‐COF particles at varying concentrations (10, 20, 50, 100, and 200 µg mL^−1^). After 24, 48, and 72 h of incubation with CS‐UCNPs and UCNP‐COF particles, the cell viability was assessed using CellTiter‐Glo assay (Promega), with untreated cells serving as 100% viability reference. All experiments were conducted in triplicate and average cell viability was obtained.

### ICG, DOX, and Insulin Loading into UCNP‐COF and Release Tests

UCNP‐COF particles were dispersed in phosphate buffer saline (PBS) at a concentration of 100 µg mL^−1^ and mixed with 500 µg mL^−1^ of indocyanine green (ICG, TCI Chemicals), 200 µg mL^−1^ of doxorubicin hydrochloride (suitable for fluorescence, Sigma–Aldrich), and 200 µg mL^−1^ of FITC‐labeled insulin (Sigma–Aldrich), respectively. Each solution was stirred at 350 rpm in the dark for 24 h to allow adsorption of ICG, DOX, and insulin. After 24 h, the suspension was centrifuged at 1000*g* three times, and the collected precipitates were used for testing. For the pH‐driven release tests of DOX and insulin, the pH of the PBS solution was adjusted using acetic acid. The amounts of released DOX and insulin were determined through UV–Vis spectroscopy measurements, which were analyzed by plotting with calibration curves over time.

### Fabrication of Magnetic Janus UCNP‐COF Particles

Magnetic Janus UCNP‐COF particles were fabricated by sequentially sputtering 50 nm of Ni and 20 nm of Au onto pre‐dried UCNP‐COF particles using a sputter coating system (Leica EM ACE600, Leica Microsystems). For preparation, the UCNP‐COF particles were dispersed in deionized water with 500 µg mL^−1^ concentration and 0.5 mL of the particle suspension was drop‐cast onto an O_2_ plasma‐treated 2 × 4 cm^2^ glass slide. After drying overnight at ambient conditions, the Ni and Au layers were deposited on the particles using the sputter system. The magnetization of the Janus particles was programmed in the out‐of‐plane direction by applying a 1.8 T uniform magnetic field using a vibrating sample magnetometer (MicroSense, Lowell, MA). Afterward, the glass slide was placed in a plastic petri dish filled with ethanol and sonicated, allowing the particles to transfer into the ethanol phase within seconds. The obtained particles were then dried at 70 °C.

### In Vivo 3D MSOT and Magnetic Steering

In vivo experiments in three mice were conducted in compliance with national guidelines of the Swiss Federal Act on Animal Protection and were approved by the Cantonal Veterinary Office Zurich. One Foxn1^nu^ nude mouse and two C57BL/6 mice (Charles River Laboratories, USA) were used in the test for Figures 3, 5, and 5f, respectively. Anesthesia in the mice was induced with 5% isoflurane and maintained at 1.5−2% isoflurane during imaging with continuous monitoring of blood oxygen saturation, heart rate, and mouse body temperature (PhysioSuite, Kent Scientific, Torrington, CT). The mouse's body temperature was maintained within physiological range using a heating pad. To ensure optimal ultrasound coupling, the mouse's hair was removed using shaving cream. For precise positioning, the mouse was placed on an *x‐y‐z* manual moving stage (Thorlabs). For in vivo MSOT imaging with the custom‐made setup, the multi‐spectral illumination from a wavelength‐tunable NIR laser (Innolas Laser GmbH, Kralling, Germany) was delivered to the mouse femoral and brain vasculature through an 8 mm diameter central aperture in a handheld spherical array probe containing 512 individual detection elements, as described elsewhere.^[^ ^19^, ^40^ ^]^ Following successful anesthesia, a bolus injection of 200 µL of 500 µg mL^−1^ magnetic Janus UCNP‐COF particles was administered into the mouse's tail vein. For magnetic steering of particles or microrobots within the femoral and brain vasculature, a NdFeB neodymium magnet was positioned beneath the mouse's hindlimb and head, respectively. The mouse was euthanized immediately after the imaging procedure using Isoflurane overdose and following decapitation.

### Histological Particle Distribution Analysis by Post‐Mortem Imaging

Organs were extracted post‐mortem from mice and immediately immersed in 4% paraformaldehyde overnight. Then, the organs are equilibrated in 15% and 30% sucrose solutions prepared in 0.1 M PBS at 4 °C. After 48 h, the organs were sectioned into 200 µm‐thick slices using a cryotome (CM3050S, Leica, Germany). The slices were then mounted on glass slides and imaged using a custom‐built two‐photon laser scanning microscope^[^ ^48^ ^]^ equipped with a tunable pulsed laser (Chameleon Discovery TPC, Coherent Inc.) and a 25× water‐immersion objective (W Plan‐Apochromat 25×/1.05 NA, Olympus). In microscopy imaging, excitation wavelengths ranged from 700 nm, and fluorescence emission signals were detected using GaAsP photomultiplier modules (Hamamatsu Photonics). The signals were filtered through 475/64, 535/50, and 607/70 nm band‐pass filters and separated by 506 or 560 nm dichroic mirrors (BrightLine, Semrock). The microscope was controlled using a customized version of ScanImage (r3.8.1, Janelia Research Campus). To distinguish the fluorescence signal of ICG@UCNP‐COF particles from tissue autofluorescence, the excitation wavelength was switched between 780 and 900 nm.

### In Vitro Magnetic Steering and MSOT Imaging of DOX‐Loaded Microrobots

In vitro artificial vascular channels within an agar phantom were fabricated by pouring an agar aqueous gel (1.4 wt.% agar) into a 3D‐printed channel mold and allowing it to solidify for 12 h. HeLa cells (American Type Culture Collection, ATCC) were seeded at a density of 1 × 10⁴ cells per well and incubated in 96‐well plates filled with DMEM supplemented with 10% FBS and 1% penicillin–streptomycin solution. After 24 h of incubation, the HeLa cells were transferred to the one end of the channel. DOX‐loaded microrobots (DOX@MRs, 100 µg mL^−1^) were injected into the opposite end of the channel, and guided toward the HeLa cells plate using a NdFeB magnet while being monitored with the imaging probe of the inVision 512‐echo imaging system.

### Cytotoxicity Test Using HeLa Cells

Ni─Au‐sputtered Janus UCNP‐COF particles (referred to as MRs) and DOX@MRs, dispersed in DMEM at varying concentrations, were added to each well containing HeLa cells and further incubated for 6, 12, and 24 h at 37 °C in a 5% CO₂ environment. Fluorescent cell images were obtained using a live/dead assay. Cells on the samples were washed twice with PBS, stained for 30 s with fluorescein diacetate/propidium iodide (FDA/PI, Thermo Fisher), and observed using a fluorescence‐inverted microscope (TS2, Nikon) at 490 nm and 550 nm to visualize the live and dead cells, respectively. To generate HeLa cell spheroids, a HeLa cell suspension (5 × 10^4^ cells per well in a 96‐well plate) was prepared. 25 µL of suspension drops were seeded on the inner side of a Petri dish lid, while the bottom of the Petri dish was filled with 25 mL of sterile distilled water. After 48 h of incubation at 37 °C in a 5% CO_2_ environment, 3D spheroids were formed within the cell suspension drops. For the MTT cytotoxicity assay, cells (1 × 10^4^ cells per well in a 96‐well plate) were treated with different concentrations (0, 10, 25, 50, 75, 100 µg mL^−1^) of the agents for 12 and 24 h. Subsequently, 25 µL of PBS containing 5 mg mL^−1^ 3‐[4,5‐dimethylthiazol‐2‐yl]‐2,5‐diphenyltetrazolium bromide (MTT, Sigma–Aldrich) was added, and the cells were incubated at 37 °C, 5% CO_2_. After 2 h of incubation, the medium was replaced with 100 µL DMSO to dissolve the formazan crystals. The plates were incubated for an additional 1 h at room temperature in the dark, and absorbance at 570 nm was measured using a microplate reader (Wallac 1420 Multilabel Counter, Perkin Elmer).

## Conflict of Interest

The authors declare no conflict of interest.

## Author Contributions

D.W.K., P.W., and A.R.C. contributed equally to this work. D.W.K. and M.S. conceived the project and designed the experiments. P.W. and A.R.C. performed the experiments and contributed to the data analysis and management. Y.C. contributed to the animal experiments. N.O.D. was involved in the cell cytotoxicity test. C.G. contributed to the two‐photon imaging of post‐mortem organs. M.S., D.R., and B.V.L. supervised the study. D.W.K. wrote the main manuscript, and all authors contributed to the manuscript editing.

## Supporting information



Supporting Information

Supplemental Movie 1

Supplemental Movie 2

Supplemental MovieS3

## Data Availability

The data that support the findings of this study are available from the corresponding author upon reasonable request.

## References

[1] S. Manohar , D. Razansky , Adv. Opt. Photonics 2016, 8, 586.

[2] X. L. Deán‐Ben , S. Gottschalk , B. Mc Larney , S. Shoham , D. Razansky , Chem. Soc. Rev. 2017, 46, 2158. 28276544 10.1039/c6cs00765a PMC5460636

[3] Z. Chen , I. Gezginer , Q. Zhou , L. Tang , X. L. Deán‐Ben , D. Razansky , Chem. Soc. Rev. 2024, 53, 6068. 38738633 10.1039/d3cs00565h PMC11181994

[4] L. Lin , L. V. Wang , Nat. Rev. Clin. Oncol. 2022, 19, 365. 35322236 10.1038/s41571-022-00615-3

[5] S. Wang , J. Lin , T. Wang , X. Chen , P. Huang , Theranostics 2016, 6, 2394. 27877243 10.7150/thno.16715 PMC5118603

[6] J. Weber , P. C. Beard , S. E. Bohndiek , Nat. Methods 2016, 13, 639. 27467727 10.1038/nmeth.3929

[7] Y. S. Chen , Y. Zhao , S. J. Yoon , S. S. Gambhir , S. Emelianov , Nat. Nanotechnol. 2019, 14, 465. 30833692 10.1038/s41565-019-0392-3 PMC6506383

[8] Q. Fu , R. Zhu , J. Song , H. Yang , X. Chen , Adv. Mater. 2019, 31, 1805875. 10.1002/adma.201805875 30556205

[9] S. Jiang , J. Lin , P. Huang , Adv. Healthcare Mater. 2023, 12, 2202208.

[10] Y. Yang , J. Huang , W. Wei , Q. Zeng , X. Li , D. Xing , B. Zhou , T. Zhang , Nat. Commun. 2022, 13, 3149. 35672303 10.1038/s41467-022-30713-w PMC9174188

[11] B. Wang , Q. Zhao , N. M. Barkey , D. L. Morse , H. Jiang , Med. Phys. 2012, 39, 2512. 22559621 10.1118/1.3700401 PMC4108689

[12] D. H. Li , B. D. Smith , Chem. ‐ Eur. J. 2021, 27, 14535.34403531

[13] S. Mindt , I. Karampinis , M. John , M. Neumaier , K. Nowak , Photochem. Photobiol. Sci. 2018, 17, 1189. 30028469 10.1039/c8pp00064f

[14] D. W. Kim , P. Wrede , H. Estrada , E. Yildiz , J. Lazovic , A. Bhargava , D. Razansky , M. Sitti , Adv. Mater. 2024, 36, 2404514. 39400967 10.1002/adma.202404514 PMC11636169

[15] Z. Chaudhary , G. M. Khan , M. M. Abeer , N. Pujara , B. W. C. Tse , M. A. McGuckin , A. Popat , T. Kumeria , Biomater. Sci. 2019, 7, 5002. 31617526 10.1039/c9bm00822e

[16] K. Gowsalya , V. Yasothamani , R. Vivek , Nanoscale Adv 2021, 3, 3332. 36133722 10.1039/d1na00059d PMC9418715

[17] Y. Sun , W. Feng , P. Yang , C. Huang , F. Li , Chem. Soc. Rev. 2015, 44, 1509. 25113504 10.1039/c4cs00175c

[18] V. Bastos , P. Oskoei , E. Andresen , M. I. Saleh , B. Rühle , U. Resch‐Genger , H. Oliveira , Sci. Rep. 2022, 12, 3770. 35260656 10.1038/s41598-022-07630-5 PMC8904531

[19] P. Wrede , O. Degtyaruk , S. K. Kalva , X. L. Deán‐Ben , U. Bozuyuk , A. Aghakhani , B. Akolpoglu , M. Sitti , D. Razansky , Sci. Adv. 2022, 8, eabm9132. 35544570 10.1126/sciadv.abm9132 PMC9094653

[20] M. K. Gnanasammandhan , N. M. Idris , A. Bansal , K. Huang , Y. Zhang , Nat. Protoc. 2016, 11, 688. 26963631 10.1038/nprot.2016.035

[21] L. Tan , D. Li , L. Zhang , L. Xu , Y. Zhao , L. Zhu , R. Qiao , J Phys Chem 2020, 124, 18081.

[22] S. Melle , O. G. Calderón , M. Laurenti , D. Mendez‐Gonzalez , A. Egatz‐Gómez , E. López‐Cabarcos , E. Cabrera‐Granado , E. Díaz , J. Rubio‐Retama , J Phys Chem 2018, 122, 18751. 10.1039/c9nr02039j 31294740

[23] W. Zheng , P. Huang , Z. Gong , D. Tu , J. Xu , Q. Zou , R. Li , W. You , J. C. G. Bünzli , X. Chen , Nat. Commun. 2018, 9, 3462. 30150637 10.1038/s41467-018-05947-2 PMC6110834

[24] D. W. Kim , J. Jang , J. W. Leem , H. Yun , B. Ko , I. S. Kim , H. Park , Y. L. Kim , J. Rho , U. Jeong , Adv. Opt. Mater. 2024, 12, 2401779.

[25] S. K. Maji , S. Sreejith , J. Joseph , M. Lin , T. He , Y. Tong , H. Sun , S. W. Yu , Y. Zhao , Adv. Mater. 2014, 26, 5633. 24913756 10.1002/adma.201400831

[26] S. He , J. Song , J. Liu , L. Liu , J. Qu , Z. Cheng , Adv. Opt. Mater. 2019, 7, 1900045.

[27] D. Wang , W. Wei , A. Singh , G. S. He , R. Kannan , L. S. Tan , G. Chen , P. N. Prasad , J. Xia , ACS Photonics 2017, 4, 2699. 30246053 10.1021/acsphotonics.7b00399 PMC6150608

[28] H. Qin , T. Zhou , S. Yang , D. Xing , Small 2015, 11, 2675. 25656695 10.1002/smll.201403395

[29] V. Sridhar , E. Yildiz , A. Rodríguez‐Camargo , X. Lyu , L. Yao , P. Wrede , A. Aghakhani , B. M. Akolpoglu , F. Podjaski , B. V. Lotsch , M. Sitti , Adv. Mater. 2023, 35, 2301126. 37003701 10.1002/adma.202301126 PMC11475396

[30] P. Bilalis , L. A. Tziveleka , S. Varlas , H. Iatrou , Polym. Chem. 2016, 7, 1475.

[31] N. Mokhtari , M. Dinari , F. K. Esmaeiltarkhani , ACS Omega 2023, 8, 25565. 37483239 10.1021/acsomega.3c03316 PMC10357574

[32] D. Fu , L. Zhong , J. Xu , A. Mo , M. Yang , RSC Adv. 2024, 14, 20799. 38952941 10.1039/d4ra01955e PMC11215751

[33] D. Kim , E. S. Lee , K. T. Oh , Z. G. Gao , Y. H. Bae , Small 2008, 4, 2043. 18949788 10.1002/smll.200701275 PMC2582593

[34] Y. Zhao , B. G. Trewyn , I. I. Slowing , V. S. Y. Lin , J. Am. Chem. Soc. 2009, 131, 8398. 19476380 10.1021/ja901831u

[35] Y. Alapan , U. Bozuyuk , P. Erkoc , A. C. Karacakol , M. Sitti , Sci Robot 2020, 5, eaba5726. 33022624 10.1126/scirobotics.aba5726

[36] G. Go , A. Yoo , K. T. Nguyen , M. Nan , B. A. Darmawan , S. Zheng , B. Kang , C. S. Kim , D. Bang , S. Lee , K. P. Kim , S. S. Kang , K. M. Kim , S. Bang , D.‐H. Kim , J. O. Park , E. Choi , Sci. Adv. 2022, 8, eabq8545. 36399561 10.1126/sciadv.abq8545 PMC9674283

[37] D. Kalyane , N. Raval , R. Maheshwari , V. Tambe , K. Kalia , R. K. Tekade , Mater. Sci. Eng. C. 2019, 98, 1252. 10.1016/j.msec.2019.01.066 30813007

[38] M. V. Baranov , M. Kumar , S. Sacanna , S. Thutupalli , G. vanden Bogaart , Front Immunol 2021, 11, 607945. 33679696 10.3389/fimmu.2020.607945 PMC7927956

[39] G. Hong , J. C. Lee , J. T. Robinson , U. Raaz , L. Xie , N. F. Huang , J. P. Cooke , H. Dai , Nat. Med. 2012, 18, 1841. 23160236 10.1038/nm.2995 PMC3595196

[40] X. L. Deán‐Ben , J. Robin , D. Nozdriukhin , R. Ni , J. Zhao , C. Glück , J. Droux , J. Sendón‐Lago , Z. Chen , Q. Zhou , B. Weber , S. Wegener , A. Vidal , M. Arand , M. E. Amki , D. Razansky , Nat. Commun. 2023, 14, 3584. 37328490 10.1038/s41467-023-39069-1 PMC10275987

[41] J. M. Campbell , M. Gosnell , A. Agha , S. Handley , A. Knab , A. G. Anwer , A. Bhargava , E. M. Goldys , Adv. Mater. 2024, 36, 2403761. 10.1002/adma.202403761 38775184

[42] M. Longmire , P. L. Choyke , H. Kobayashi , Nanomed. 2008, 3, 703 10.2217/17435889.3.5.703 PMC3407669 18817471

[43] M. Sitti , Mobile Microrobotics, The MIT Press, Cambridge, MA, USA 2017.

[44] A. Trebinska‐Stryjewska , O. Swiech , L. J. Opuchlik , E. A. Grzybowska , R. Bilewicz , ACS Omega 2020, 5, 7979. 32309708 10.1021/acsomega.9b04479 PMC7161040

[45] D. W. Kim , Y. Hagiwara , S. Hasebe , N. O. Dogan , M. Zhang , T. Asahi , H. Koshima , M. Sitti , Adv. Funct. Mater. 2023, 33, 2305916.

[46] D. W. Kim , C. Hyun , T. J. Shin , U. Jeong , ACS Nano 2022, 16, 2521. 35044152 10.1021/acsnano.1c09140

[47] R. L. Li , N. C. Flanders , A. M. Evans , W. Ji , I. Castano , L. X. Chen , N. C. Gianneschi , W. R. Dichtel , Chem. Sci. 2019, 10, 3796. 30996969 10.1039/c9sc00289h PMC6446964

[48] J. M. Mayrhofer , F. Haiss , F. Helmchen , B. Weber , Neuroimage 2015, 115, 52. 25934471 10.1016/j.neuroimage.2015.04.045

