# Supplementary material for: Upconversion Nanoparticle‐Covalent Organic Framework Core–shell Particles as Therapeutic Microrobots Trackable With Optoacoustic Imaging
Source: Adv Mater. 2025 Mar 7;37(49):2418425. doi: 10.1002/adma.202418425 (PMC12691905; doi:10.1002/adma.202418425)
Supplement: Supplementary file 1 — Supporting Information [file ADMA-37-2418425-s001.pdf]

ADVANCED MATERIALS

Supporting Information

for *Adv. Mater.*, DOI 10.1002/adma.202418425

Upconversion Nanoparticle-Covalent Organic Framework Core–shell Particles as Therapeutic Microrobots Trackable With Optoacoustic Imaging

Dong Wook Kim, Paul Wrede, Andrés Rodríguez-Camargo, Yi Chen, Nihal Olcay Dogan, Chaim Glück, Bettina V. Lotsch, Daniel Razansky* and Metin Sitti**

Supporting Information

Upconversion Nanoparticle-Covalent Organic Framework Core-Shell Particles as Therapeutic Microrobots Trackable with Optoacoustic Imaging

Dong Wook Kim, Paul Wrede, Andrés Rodríguez-Camargo, Yi Chen, Nihal Olcay Dogan, Chaim Glück, Bettina V. Lotsch, Daniel Razansky*, and Metin Sitti**

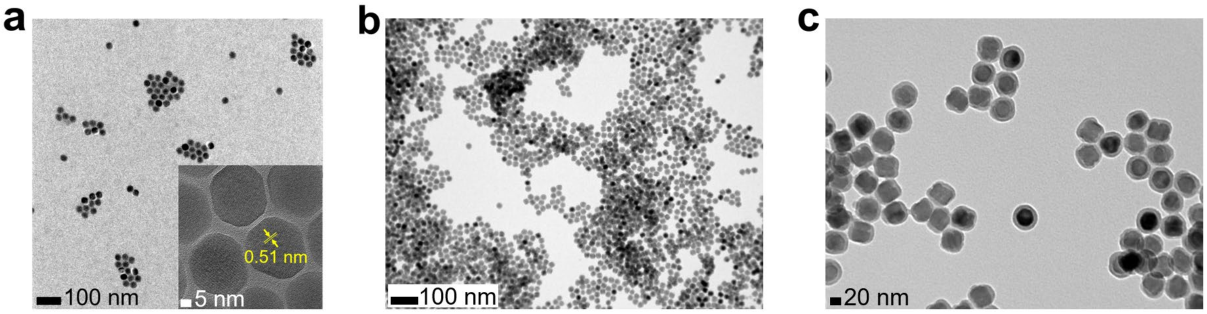

Figure S1. **Core@shell upconversion nanoparticles (CS-UCNPs).** a) Transmission electron microscopy (TEM) images of core $NaYF_4$:Yb,Tm@$NaYF_4$@$NaYF_4$:Yb,Nd UCNPs. The inset shows high-resolution TEM (HR-TEM) images displaying a lattice spacing of 0.51 nm corresponding to the (100) crystal plane of the hexagonal phase. b,c) TEM images of SiO_2 layer-coated CS-UCNPs, composed of $NaYF_4$:Yb,Tm@$NaYF_4$@$NaYF_4$:Yb,Nd@SiO_2 layers.

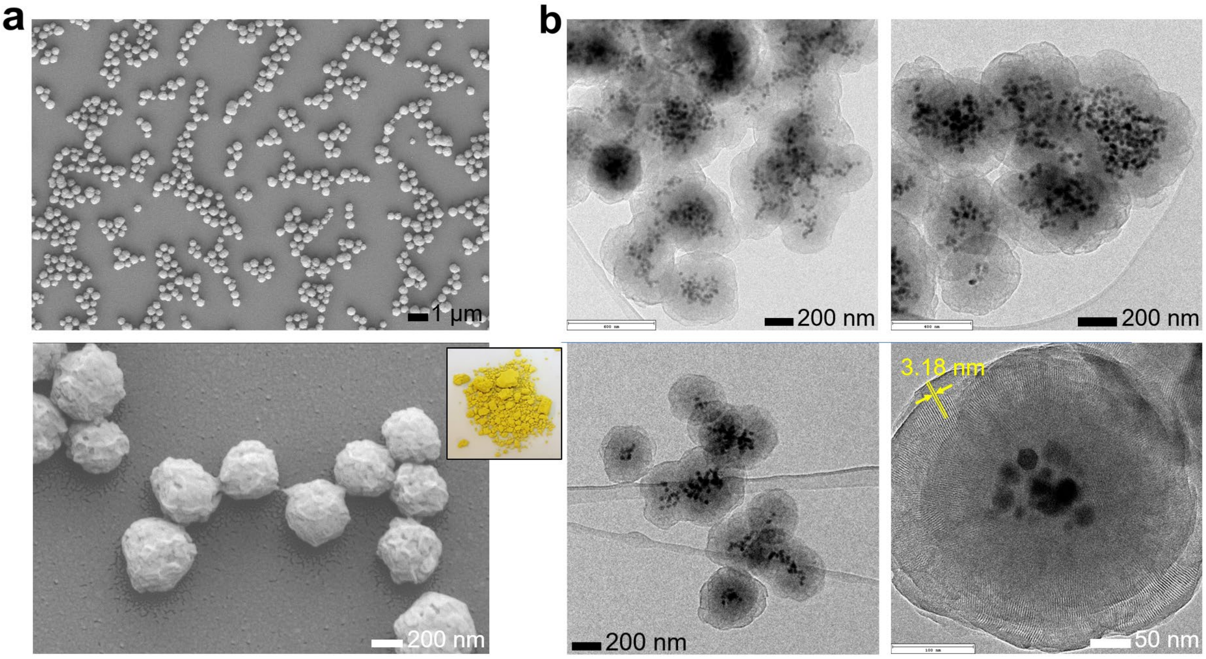

Figure S2. Upconversion nanoparticle-covalent organic framework (UCNP-COF) core-shell particles. a) Scanning electron microscopy (SEM) images of UCNP-COF sub-micron particles. The inset photograph shows the dried UCNP-COF particles. b) HR-TEM images of UCNP-COF particles, showing encapsulated CS-UCNPs within the ordered mesoporous matrix of TAPB-TPA COF.

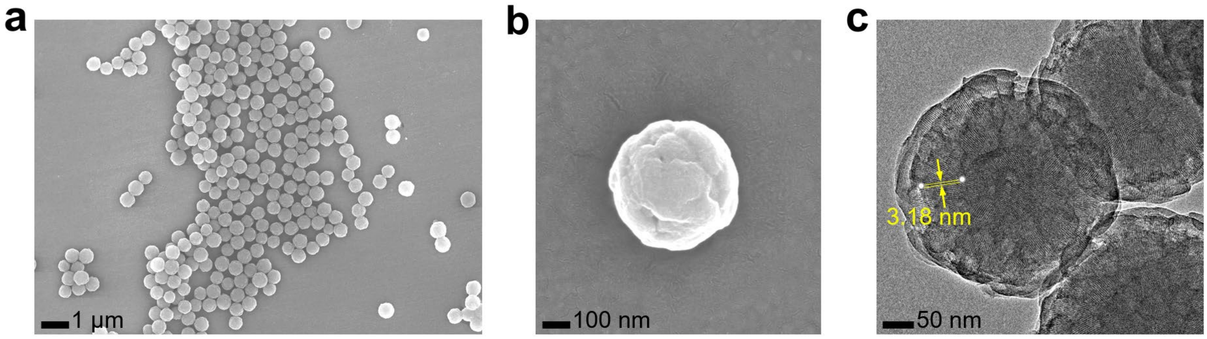

Figure S3. Pristine TAPB-TPA COF particles without CS-UCNPs. a,b) SEM images of pristine TAPB-TPA COF particles. c) HR-TEM image showing the ordered mesoporous structure of TAPB-TPA COF particles.

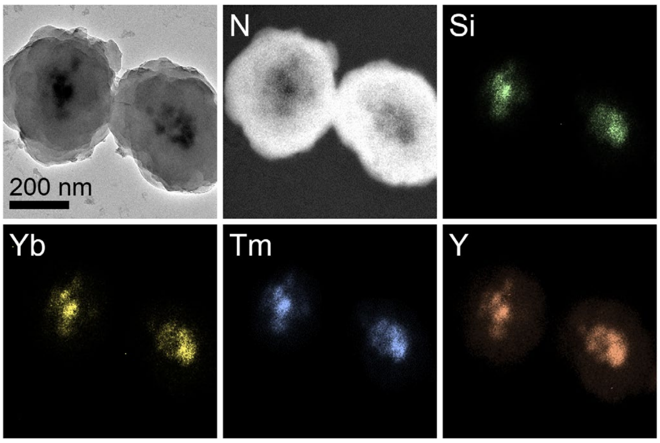

Figure S4. Elemental analysis of UCNP-COF particles. Electron energy loss spectroscopy (EELS)-assisted elemetal mapping of UCNP-COF particles.

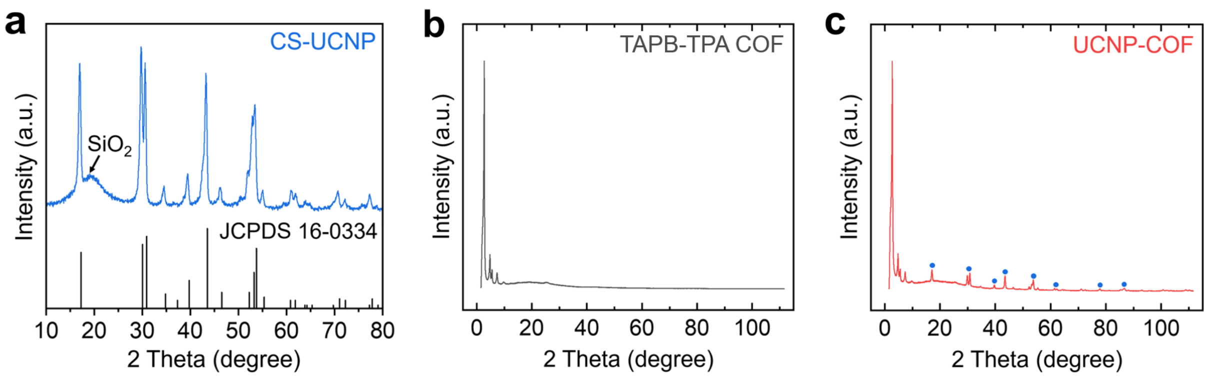

Figure S5. X-ray diffraction (XRD) studies on various samples. a–c) XRD patterns of CS-UCNPs compared with standard data (JCPDS No. 16-0334) (a), pristine TAPB-TPA COF (b), and UCNP-COF (c) particles. The blue dots in (c) correspond to the diffraction peaks of the CS-UCNPs.

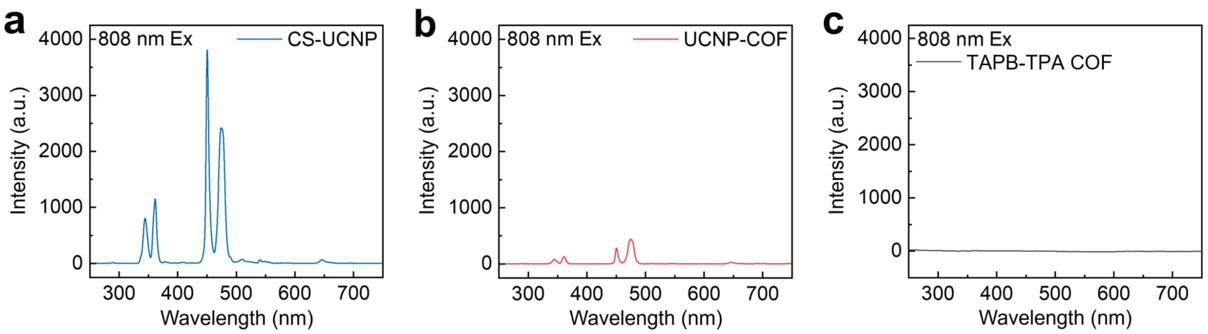

Figure S6. Luminescent characteristics of different samples. a–c) Fluorescence spectra of CS-UCNPs (a), UCNP-COF particles (b), and TAPB-TPA COF particles (c) under continuous-wave 808 nm NIR laser excitation (Ex).

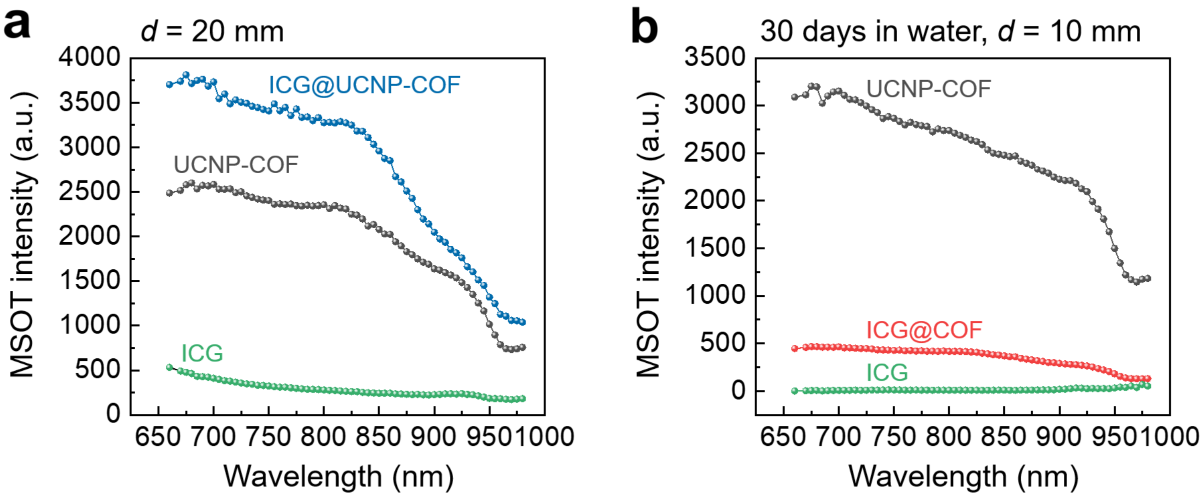

Figure S7. Multispectral optoacoustic tomography (MSOT) spectra of samples. a) MSOT spectra of ICG@UCNP-COF, UCNP-COF, and ICG samples embedded in an agar phantom at a depth (d) of 20 mm. b) MSOT spectra of UCNP-COF, ICG@COF, and ICG samples after 30 days of aging in water under white light exposure. These spectra correspond to the samples shown in Figure 3f.

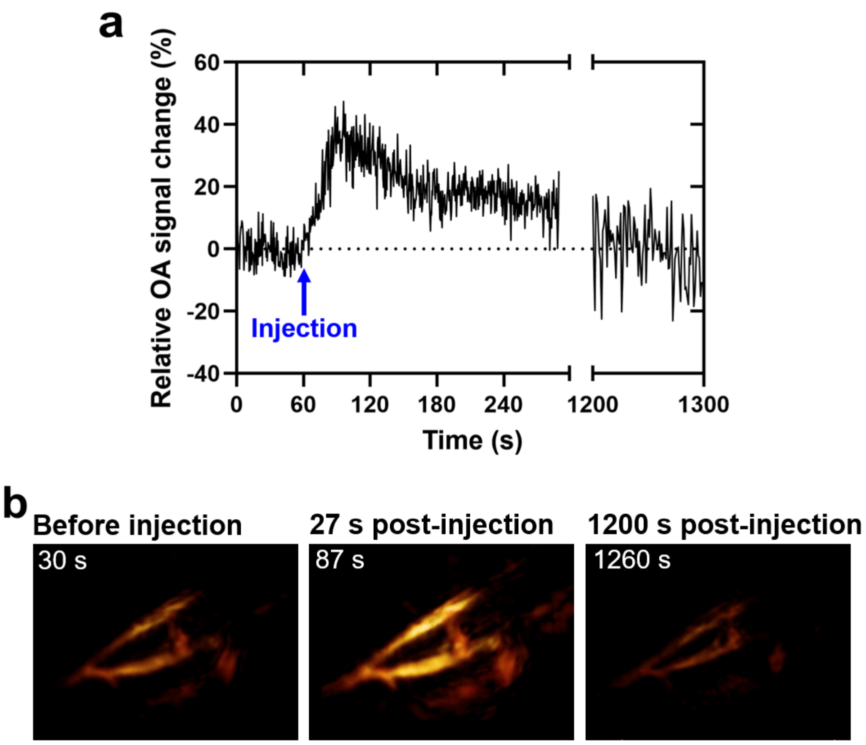

Figure S8. Time-dependent changes in MSOT intensity of the femoral vasculature following the injection of UCNP-COF particles over time. a,b) Graph showing the relative OA signal intensity over 21 min MSOT imaging. The UCNP-COF particles stored in water for 4 months were injected into the mice femoral vasculature at 1-min time point. (b) MSOT imaging results at different time points.

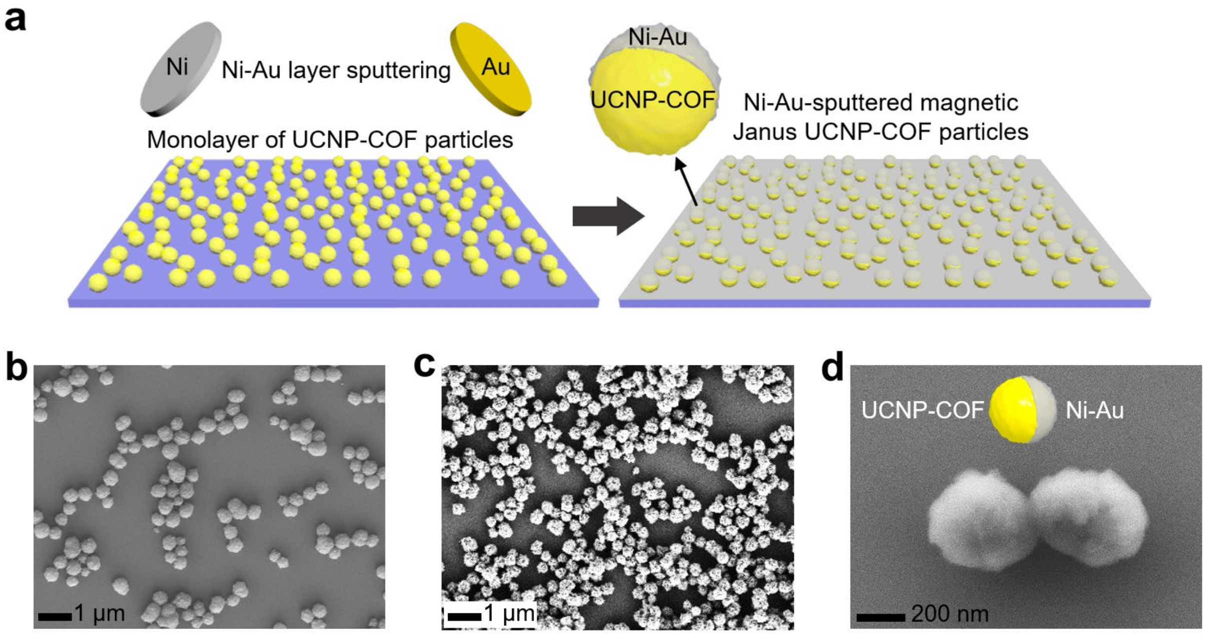

Figure S9. Ni-Au-sputtered Janus UCNP-COF particles. a) Schematic fabrication process of magnetic Janus UCNP-COF particles. b,c) SEM image of the monolayered UCNP-COF particles on a glass film before (b) and after Ni-Au sputtering (c). d) SEM image of the magnetic Jauns UCNP-COF particles.

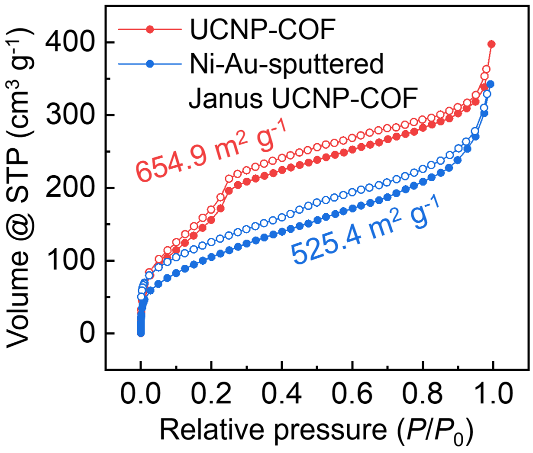

Figure S10. **Comparison of surface areas between UCNP-COF and magnetic Janus UCNP-COF particles.** Surface areas of UCNP-COF particles and Ni-Au-sputtered Janus UCNP-COF particles, measured using N_2 adsorption-desorption isotherms.

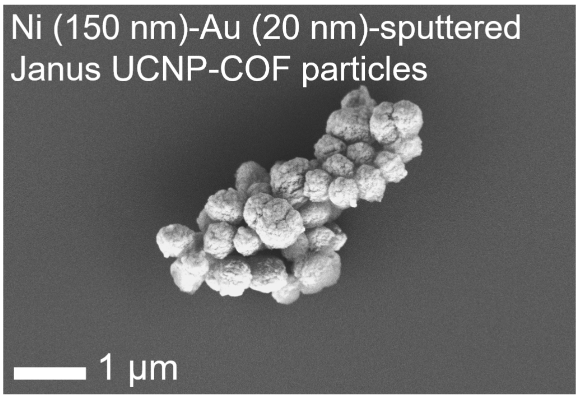

Figure S11. Thick Ni layer-sputtered Janus UCNP-COF particles. SEM image of the Ni (150 nm-thick)-Au (20 nm-thick)-sputtered Janus UCNP-COF particles. Relatively thick Ni layer-sputtered Janus particles tended to agglomerate due to their strong magnetic properties.

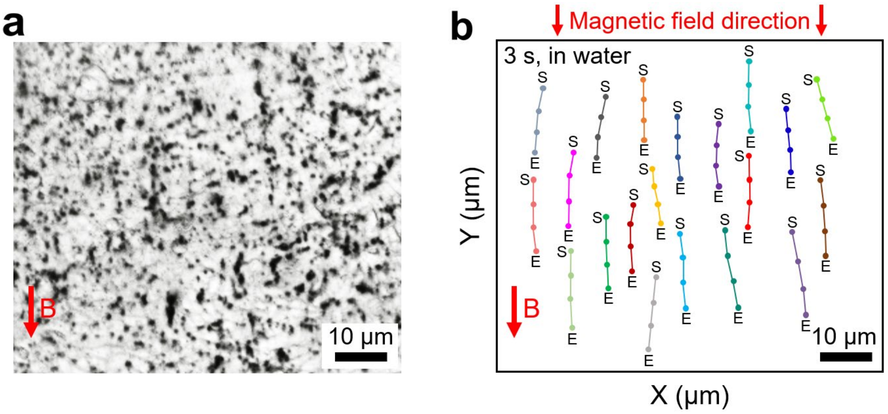

Figure S12. **Magnetic Janus UCNP-COF particles under a directional magnetic field.** a) Optical microscopy image of magnetic Janus UCNP-COF particles guided by a magnetic field in deionized water. b) Motion tracking of magnetic UCNP-COF particles shown in (a) over 3 s (S = start, E = end of trajectory).

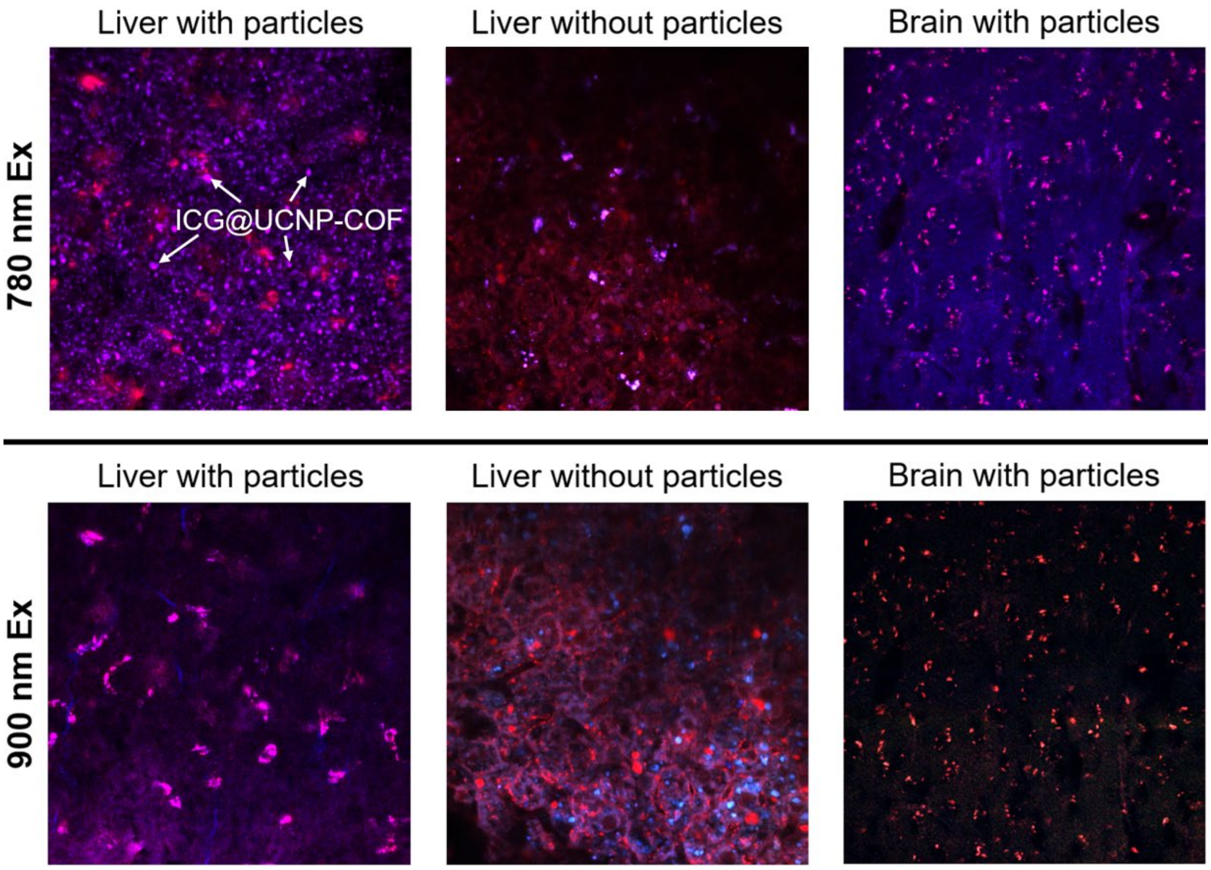

Figure S13. Two-photon microscopy images of sliced post-mortem liver and brain tissues under 780 nm and 900 nm excitation (Ex). In mice injected with ICG@UCNP-COF particles, which are excited primarily by 780 nm wavelength, the liver tissue exhibited significant differences between 780 nm and 900 nm imaging, attributed to the presence of particles cleared by the liver. In contrast, the liver tissue from non-treated mice and the brain tissue from particle-injected mice showed no noticeable differences at either wavelength.

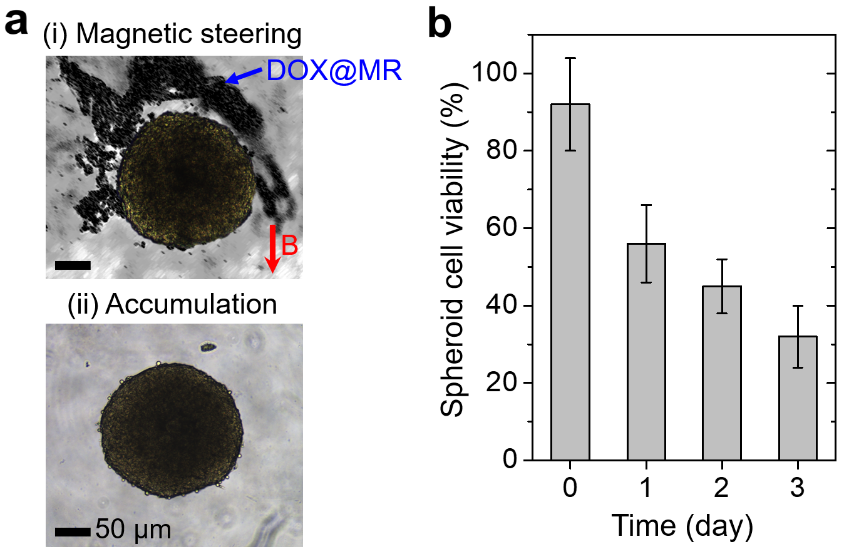

Figure S14. Magnetic steering of DOX@MRs to three-dimensioanl HeLa spheroids and their therapeutic performace. a) Microscopy images showing the magnetic steering of DOX@MRs toward a HeLa spheroid and their accumulation onto the spheroid. b) Cell viability of the spheroid assessed over 3 days.

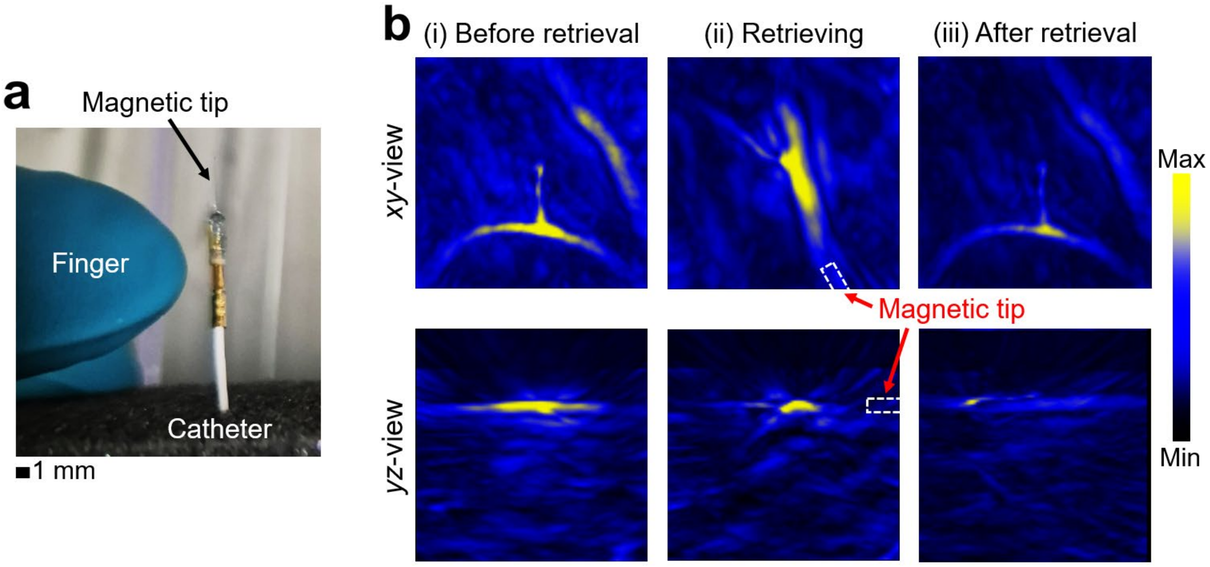

Figure S15. Magnetic retrieval of magnetic Janus UCNP-COF particles using a magentic tip-attached catheter. a) Digital photograph of the catheter with a magnetic tip. b) MSOT imaging of the phantom vessels before retrieval (i), retrieving moment of the magnetic UCNP-COF particles (ii), and after retrieval.

Supporting Movies

Movie S1. In vivo 3D MSOT imaging of UCNP-COF particles injected into mouse brain vasculature

Movie S2. In vivo 3D MSOT imaging of magnetic Janus UCNP-COF particles in mouse femoral vasculature

Movie S3. In vivo 3D MSOT imaging of magnetic Janus UCNP-COF particles in mouse brain vasculature
